# Anti-Diabetic Potential of Polyphenol-Rich Fruits from the Maleae Tribe—A Review of In Vitro and In Vivo Animal and Human Trials

**DOI:** 10.3390/nu15173756

**Published:** 2023-08-28

**Authors:** Magdalena Rutkowska, Monika A. Olszewska

**Affiliations:** Department of Pharmacognosy, Faculty of Pharmacy, Medical University of Lodz, 1 Muszynskiego St., 90-151 Lodz, Poland; monika.olszewska@umed.lodz.pl

**Keywords:** Maleae fruits, diabetes, glucose transport, insulin signalling pathway, carbohydrate digestion, polyphenols, polysaccharides, triterpenes

## Abstract

The Maleae tribe consists of over one thousand species, including many well-known polyphenol-containing fruit crops with wide-ranging biological properties, e.g., apples (*Malus*), chokeberries (*Aronia*), pears (*Pyrus*), quinces (*Cydonia*, *Chaenomeles*), saskatoon (*Amelanchier*), loquats (*Eriobotrya*), medlars (*Mespilus*), rowans (*Sorbus*), and hawthorns (*Crataegus*). Considering the current interest in the concept of functional foods and the still-insufficient methods of diabetes management, the anti-diabetic potential of fruits has been studied intensively, including those of the Maleae tribe. This paper is the first comprehensive overview of this selected topic, covering articles published from 2000 to 2023 (131 articles in total). The first part of this review focuses on the potential mechanisms of action of fruits investigated so far (46 species), including their effects on tissue-specific glucose transport and the expression or activity of proteins in the insulin signalling pathway. The second part covers the phytocompounds responsible for particular fruits’ activity—primarily polyphenols (e.g., flavonols, dihydrochalcones, proanthocyanidins, anthocyanins, phenolic acids), but also polysaccharides, triterpenes, and their additive and synergistic effects. In summary, fruits from the Maleae tribe seem promising as functional foods and anti-diabetic agents; however, their prospects for more expansive pro-health application require further research, especially more profound in vivo trials.

## 1. Introduction

Diabetes is a chronic, progressive disorder characterised by raised blood glucose levels due to insufficient production of the hormone insulin or decreased effectiveness of the insulin that the body produces [1,2]. Long-term hyperglycaemia has become a significant healthcare burden worldwide, leading to life-threatening damage to the blood vessels, heart, nerves, and kidneys [2]. It was estimated that 353 million patients suffered from diabetes in 2021, and it is projected that by 2030 there will be about 643 million people diagnosed with this disorder [1]. Therefore, diabetes has been recognised as one of four non-communicable diseases targeted as a priority by the world’s public health leaders [2].

The pharmacotherapy of diabetes includes insulin (when insulin deficiency is seen) or oral hypoglycaemic drugs that exert anti-diabetic effects through different mechanisms. These mechanisms comprise, i.a., stimulation of endogenous insulin secretion from pancreatic β-cells by sulfonylureas, glucagon-like peptide 1 (GLP-1) analogues, or dipeptidyl peptidase-4 (DPP IV) inhibitors; the increase in the insulin sensitivity, the boost of peripheral absorption of glucose, and the reduction in hepatic gluconeogenesis by peroxisome proliferator-activated receptor γ (PPARγ) activators or biguanides; the delay in the absorption of carbohydrates from the intestine by α-glucosidase inhibitors; or the increase in glucose elimination via the kidneys by sodium–glucose cotransporter-2 (SGLT2) inhibitors [3,4,5]. However, the conventional treatment of diabetes often fails due to drug resistance (reduction in efficiency over time) and, perhaps even more critically, various side effects and, thus, noncompliant behaviour of patients. Therefore, a constant search is underway for better diabetes management, including lifestyle modifications, proper diet, and the use of medicinal plants [2,3,4].

The anti-diabetic potential of fruits and vegetables has been widely studied, as the concept of functional foods (i.e., dietary products that offer health benefits beyond their nutritional value) has gained importance [6,7]. Research on the anti-hyperglycaemic effects of fruits has revealed their involvement in glucose transport and metabolism through mechanisms of action that seem analogous to synthetic anti-diabetic drugs. These mechanisms include, e.g., the modulation of glucose transporters’ activity, the inhibition of carbohydrate digestion, or multiple effects on glycolysis, gluconeogenesis, glycogen synthesis, and glycogenolysis by affecting the activity or expression of proteins in the insulin signalling pathway [7]. Thus, it has been suggested that many dietary fruits might be used not only in the prevention of diabetes, but also as a potential source of new anti-diabetic agents for mono- or combination therapy, enabling the reduction in synthetic drug doses and, thus, the side effects of conventional diabetes treatment [6].

The Maleae tribe comprises over one thousand species, mainly from the Northern Hemisphere [8]. It includes many well-known fruit crops, e.g., apples (*Malus* Mill. sp.), pears (*Pyrus* L. sp.), black chokeberry (*Aronia melanocarpa* (Michx.) Elliott), quince (*Cydonia oblonga* Mill.) and Japanese quince (*Chaenomeles japonica* (Thunb.) Lindl. ex Spach), saskatoon berry (*Amelanchier alnifolia* Nutt.), loquat (*Eriobotrya japonica* (Thunb.) Lindl.), medlar (*Mespilus germanica* L.), rowans (*Sorbus* L. sp.), and hawthorns (*Crataegus* L. sp.). Fruits from Maleae species are widely consumed both unprocessed and as jams, juices, alcoholic beverages (e.g., wines, liqueurs), etc. [9]. According to the United States Department of Agriculture [10], the global annual consumption of only fresh apples and pears was 81.6 and 23.5 Mt in 2021, respectively, making them some of the most willingly consumed fruits in the world. Consequently, the pro-health properties of fruit consumption have been intensively studied in recent decades [6], including their anti-hyperglycaemic activity.

This paper presents the first comprehensive overview of the anti-diabetic potential of the fruits of the Maleae tribe. It covers articles published from 2000 to 2023, including in vitro research, in vivo tests on animal models, and in vivo human trials. The main focus of this review was to indicate the biological effectiveness of the fruits investigated so far, discuss the potential mechanisms of action behind the observed effects, and point out the phytocompounds that may be responsible for the anti-diabetic properties of different Maleae fruits. Apart from summarising the current knowledge and challenges, we discuss the further research directions required for a deeper understanding of the biological properties and potential of Maleae fruits for their wider use as functional products or anti-diabetic phytotherapeutics.

## 2. Materials and Methods

The literature selection was performed based on the Scopus, Web of Science, and Google Scholar databases, searching for original articles written in English and published (at least electronically) between January 2000 and June 2023. The search was conducted using the following keyword combination pattern: (1) the genus Latin or common nomenclature (i.e., “*Amelanchier*” or “*Amelasorbus*” or “*Aronia*” or “*Chaenomeles*” or “*Chamaemeles*” or “*Cotoneaster*” or “*Crataegus*” or “*Crataemespilus*” or “*Cydonia*” or “*Dichotomanthes*” or “*Docynia*” or “*Eriobotrya*” or “*Eriolobus*” or “*Hesperomeles*” or “*Heteromeles*” or “*Kageneckia*” or “*Lindleya*” or “*Malacomeles*” or “*Malus*” or “*Mespilus*” or “*Osteomeles*” or “*Peraphyllum*” or “*Photinia*” or “*Pseudocydonia”* or “*Pyracantha*” or “*Pyrus*” or “*Rhaphiolepis*” or “*Sorbus*” or “*Sorbaronia*” or “*Sorbocotoneaster*” or “*Stranvaesia*” or “*Vauquelinia*” or “saskatoon” or “chokeberry” or “hawthorn” or “quince” or “apple” or “crabapple” or “medlar” or “pear” or “loquat” or “rowan” or “service tree” or “whitebeam” or “toyon”); (2) description of the plant part/product (i.e., “fruit/-s” or “berry/-ies” or “juice/-s” or “extract/-s”); (3) activity descriptor (i.e., “diabetes” or “diabetic” or “anti-diabetic” or “glucose” or “insulin” or “glycaemia/glycemia”). Only articles covering the topic of fruits’ effects on carbohydrate bioavailability/metabolism and direct toxic effects of hyperglycaemia (e.g., AGE formation) were included. Consequently, the studies on the conditions accompanying or resulting from diabetes as an outcome of complex mechanisms (e.g., inflammation, neurodegenerative diseases or cardiovascular complications of diabetes) were not reviewed. Moreover, if the paper included the analysis of both diabetic and diabetes comorbid-disorder-related parameters (e.g., lipid profiles, cytokine levels, etc.), only the first part was included in this review. The further exclusion criteria were as follows: papers covering only ethnobotanical research on plants used in diabetes (without activity studies), the effects of a complex diet or combination of plants/drugs (making it impossible to indicate which component determines the activity), and studies of single, isolated compounds of plant origin, not covering the activity or health impact of whole fruits. The inclusion or exclusion of the articles was validated manually by reading the entire item. The binomial names of the reviewed species were checked and revised according to World Flora Online [11].

## 3. Results and Discussion

**Figure 1:**
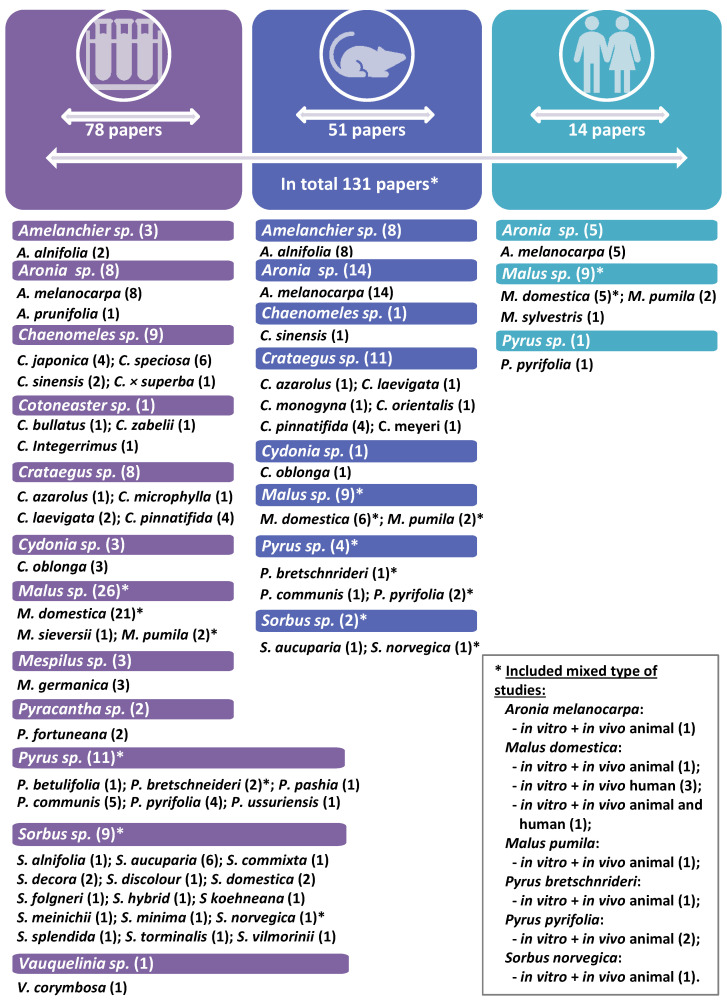
The list of species included in the review, divided into those tested in vitro, in vivo on animal models, and in vivo on human participants. Numbers in parentheses indicate the number of papers on the relevant genus/species (some papers discussed different species, and in some cases there was no indication of the exact species).

As a result of an in-depth analysis of the literature data covering articles published from 2000 to 2023, 131 studies were included in the present review. The majority of them covered only in vitro tests (67 papers); then, there were in vivo studies on animal models (51 items, including some mixed in vitro/in vivo tests) and human in vivo trials (14 papers, including one in vivo animal and human study) (Figure 1). The selected documents were based on the activity testing of fruits from 46 species belonging to the genera *Amelanchier*, *Aronia*, *Chaenomeles*, *Cotoneaster*, *Crataegus*, *Cydonia*, *Malus*, *Mespilus*, *Pyracantha*, *Pyrus, Sorbus*, and *Vauquelinia*. The most widely studied taxa among this group were *Aronia melanocarpa* (26 studies, including 13 animal and 5 human trials) and *Malus domestica* (26 studies, including 6 animal and 5 human trials) (Figure 1).

### 3.1. In Vitro Studies

Most in vitro studies of the anti-diabetic effects of Maleae fruits are based on testing their impact on α-glucosidase or α-amylase (Table 1). While these enzymes are involved in the chain reactions of carbohydrates’ breakdown to glucose (or fructose), inhibiting one or two of them may reduce the intestinal absorption of saccharides and, thus, lower postprandial hyperglycaemia. The reviewed data suggest that extracts or juices of fruit origin are relatively weak inhibitors of α-amylase; on the other hand, they can strongly inhibit α-glucosidase. For instance, the inhibitory effects of fruit extracts/juices of *Aronia melanocarpa* [12,13,14], *Aronia prunifolia* [13], *Cotoneaster integerrimus, Cotoneaster zabelii, Cotoneaster bullatus* [15], *Crataegus laevigata* [16], *Crataegus pinnatifida* [17], *Malus domestica* [18], *Pyracantha fortuneana* [19,20], *Pyrus pashia* [21], *Sorbus alnifolia, Sorbus folgneri, Sorbus minima, Sorbus norvegica, Sorbus hybrid, Sorbus aucuparia, Sorbus meinichii, Sorbus torminalis* [22,23], and *Vauquelinia corymbosa* [24] towards α-glucosidase were many times higher than that observed for acarbose—a known anti-diabetic drug and α-glucosidase inhibitor. The synergistic effects of the *Malus domestica* juice and *Sorbus aucuparia* extracts with acarbose were also suggested [25,26,27].

**Figure 2:**
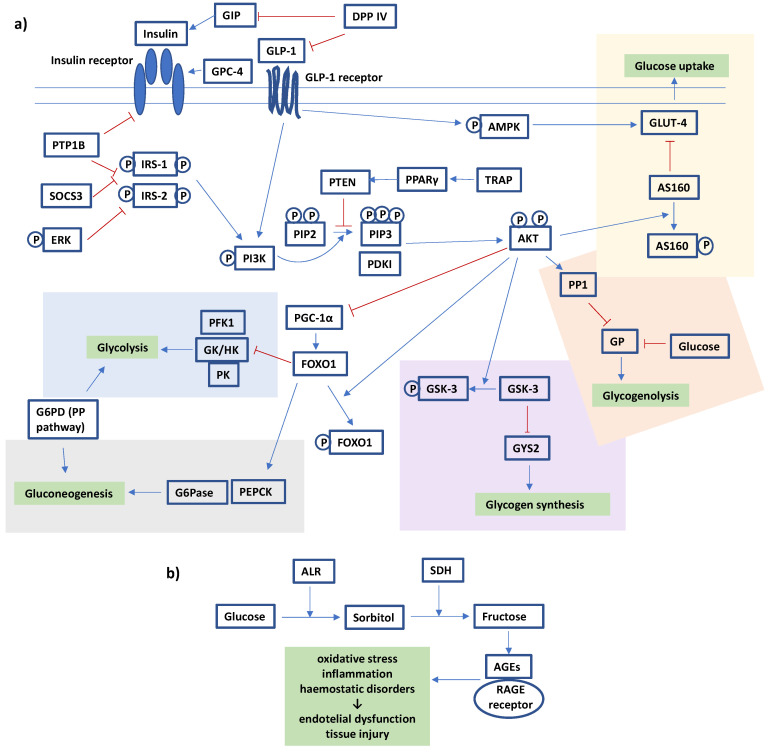
The diagram of glucose metabolism (**a**) associated with the insulin signalling pathway and (**b**) associated with the polyol pathway. The gripping points of the activity tested for different Maleae fruits are described in the manuscript body and tables. The diagram is based on data from the literature [45,46,47,48,49,50,51,52,53,54]; blue arrows indicate activation, red Ts indicate inhibition, “P” indicates phosphorylation. Abbreviations: AGEs, advanced glycation end products; AKT, protein kinase B; ALR, aldose reductase; AMPK, AMP-activated protein kinase; AS160, AKT substrate of 160 kDa; DPP IV, dipeptidyl peptidase IV; ERK, extracellular signal-regulated kinases; FOXO1, forkhead box G1; G6Pase, glucose-6-phosphatase; G6PD, glucose-6-phosphate dehydrogenase; GIP, glucose-dependent insulinotropic polypeptide; GK, glucokinase; GLP-1, glucagon-like peptide 1; GLUT-4, glucose transporter-4; GP, glycogen phosphorylase; GPC-4, glypican-4; GSK-3, glycogen synthase kinase 3; GYS2, glycogen synthase 2; HK, hexokinase; IRS-1, insulin receptor substrate 1; IRS-2, insulin receptor substrate 2; PDK1, phosphoinositide-dependent kinase 1; PEPCK, phosphoenolpyruvate carboxykinase; PFK1, phosphofructokinase 1; PGC-1α, peroxisome proliferator-activated receptor gamma, coactivator 1 alpha; PI3K, phosphoinositide 3-kinase; PIP2, phosphatidylinositol 4,5-bisphoshate; PIP3, phosphatidylinositol 3,4,5-triphoshate; PK, pyruvate kinase; PP pathway, pentose phosphate pathway; PP1, protein phosphatase 1; PPARγ, peroxisome proliferator-activated receptor gamma; PTEN, phosphatase and tensin homolog; PTP1B, protein tyrosine phosphatase 1B; RAGE receptor, receptor for advanced glycation end products; SDH, sorbitol dehydrogenase; SOCS3, suppressor of cytokine signalling 3; TRAP, mediator of RNA polymerase II transcription subunit.

The second most frequent type of in vitro research into Maleae fruits involves cellular studies on glucose transport and metabolism (Table 1). Stimulating glucose uptake through skeletal muscle or hepatic cells is one of the mechanisms that may lead to the enhancement of glucose metabolism and reduction in blood sugar levels. This effect, associated with the increased expression of insulin-dependent glucose transporter 4 (GLUT-4), has been observed for *Aronia melanocarpa* [28], *Chaenomeles japonica* [29], *Malus pumila* [30], and *Pyrus pyrifolia* [31]. Moreover, the inhibition of intestinal glucose absorption (i.e., transport from the intestinal lumen into enterocytes) via, e.g., the modulation of SGLT1 (sodium–glucose transport protein-1) and GLUT-2 (glucose transporter 2) levels or activity, has been proposed for *Malus domestica* [32,33,34]. As for the metabolic part, the effects on the expression or activity of the PI3K/Akt pathway proteins (Figure 2a) were observed for *Aronia melanocarpa* [28,35], *Chaenomeles japonica* [29,36], *Crataegus pinnatifida* [17], *Malus domestica* [37,38], *Malus pumila* [30], and *Pyrus pyrifolia* [31,39]. Considering the complexity of the processes involved in insulin and glucose regulation, the results of the studies mentioned above may indicate the ability of the tested extracts to stimulate glycolysis and glycogen synthesis, inhibit gluconeogenesis and glycogenolysis, and lower blood glucose levels. Moreover, fruits from *Amelanchier alnifolia* [40], *Crataegus pinnatifida* [17], *Malus domestica* [41], *Malus sieversii* [42], *Sorbus aucuparia* [43], and *Sorbus domestica* [44] have been suggested to inhibit enzymes involved in the polyol pathway of glucose metabolism, such as aldose reductase (ALR) and sorbitol dehydrogenase (SDH), and to impair the production of advanced glycation end products (AGEs). Thus, they may prevent diabetic complications, primarily oxidative-stress-related damage to the microvascular systems, caused by pathological glucose metabolism (Figure 2b). Furthermore, one of the investigated species, i.e., *Chaenomeles japonica* [36], was proven to have cytoprotective effects on βTC3 pancreatic β-cells (model of induced toxicity), enabling their viability and normal proliferation to be preserved. Detailed information on the accumulated research can be found in Table 1.

**Table 1:** In vitro studies of the anti-diabetic potential of fruits from the Maleae tribe.

Species	Sample Type, Composition	Model, Study Design	Tested Parameters, Observed Effects *	Ref.
*Amelanchier alnifolia* Nutt.	crude 80% EtOH extract, water fraction, and EtOAc fraction; detected compounds (HPLC-MS): phenolic acids (chlorogenic, caffeic, hydroxybenzoic acids), anthocyanins (cyanidin monoglycosides, cyanidin 3,5-diglucoside), proanthocyanidins (oligomers, catechin/epicatechin, epicatechin gallate)	cellular studies (L6rat skeletal muscle cells) and in vitro enzyme inhibition; control: 3,3-tetramethylene-glutaric acid (ALR activity), cells without an extract (glycogen accumulation)	ALR inhibition: strong activity (82 ± 0.73% inhibition at 5µg/mL) for EtOAc, other extracts not active; glycogen accumulation in non-insulin-stimulated cells (glucose uptake): 76%, 92%, and 23% changes for 80% EtOH extract, water, and EtOAc fractions, respectively, compared to controls; phenolic acids, anthocyanins, and proanthocyanidins responsible for hypoglycaemic activity (based on literature studies and extracts’ composition)	[40]
	70% acetone extract of whole fruits, flesh, and peels from different cultivars; polyphenols 1.11–2.27 g GAE/100 g (fruits), 0.45–0.96 (flesh), 1.11–2.86 (peel) (spectrophotometry), the contents of monophosphate nucleotides and free amino acids were also determined (LC-MS)	in vitro enzyme inhibition; control: -	α-amylase inhibition: IC_50_ = 18.33–31.70 mg/mL (fruits), 22.53–42.15 (flesh), 11.05–18.41 (peels); α-glucosidase inhibition: IC_50_ = 27.83–42.23 mg/mL (fruits), 28.86–43.79 (flesh), 23.60–37.06 (peels); free amino acids responsible for observed effects (PCA analysis)	[55]
*Amelanchier* Medik. sp.	70% acetone extract, polyphenols 13.8–15.2 mg/g of dry fruits (depending on clone), including hydroxycinnamic acids, anthocyanins, flavonols, and hydroxybenzoic acids (HPLC)	in vitro enzyme inhibition and cellular studies (on βTC3 pancreatic β-cells); control: sodium orthovanadate (for PTP1B)	α-amylase inhibition: IC_50_ = 4.3–5.3 mg/mL depending on clone (better than, i.a., mulberry fruits); α-glucosidase inhibition: IC_50_ = 116.7–134.3 mg/mL depending on clone (very low activity); PTP1B inhibition: IC_50_ = 1.2 mg/mL (better than, i.a., mulberry fruits); cytoprotection of βTC3 pancreatic β-cells: very low effect on cell viability and no effect on cell proliferation	[36]
*Aronia melanocarpa* (Michx.) Elliott	acidified EtOH extract; anthocyanins 128.36 μg/mL (HPLC-MS: cyanidin monoglycosides)	cellular studies (HepG2 human hepatoma cell line and C2C12 mouse myoblast cell line with palmitic acid induced insulin resistance (tested) or normal cells (control)	glucose uptake: ↑, optimal extract concentration 40 μg/mL; glycogen level: ↑; protein expression: ↑ GLUT-4, ↑ IRS-1, ↑ *p*-GSK-3β, ↓ SOCS3, ↓ *p*-IRS-1, ↓ GSK-3β compared to insulin-resistant cells (effects on glucose transport and insulin sensitivity); anthocyanins responsible for observed effects (based on literature studies and extracts’ composition)	[28]
	standardised *Aronia* berry extract powder (Fort Wayne, IN, USA); polyphenols 40%, anthocyanins 15%	cellular studies (RAW 264.7 and mouse bone-marrow-derived macrophages (BMDMs)); normal or LPS-stimulated cells (control), LPS +50, 100 mg/mL extract (tested)	mRNA expression levels: ↓ GLUT-1, HK1, and G6PD in comparison to LPS-stimulated controls (to the level comparable to normal controls); anthocyanins responsible for observed effects (based on literature studies and extract composition)	[35]
	EtOH and 50% EtOH extracts; detected compounds (HPLC-MS): cyanidin monoglycosides; epicatechin; procyanidins B2, B5, and C1	in vitro enzyme inhibition; control: acarbose IC_50_ = 130 ± 20 μg/mL	α-glucosidase inhibition: EtOH inactive; 50% EtOH IC_50_ = 3.5 ± 0.1 μg/mL; cyanidin glycosides IC_50_ = 0.37–5.5 μg/mL (the highest activity for cyanidin arabinoside); procyanidins IC_50_ = 3.8–5.5 μg/mL (the highest activity for procyanidin C1)	[12]
	80% EtOH and acidified MeOH extracts; polyphenols 98–148 mg GAE/g of EtOH extract (1079–1921 mg GAE/100 g of fresh fruits), anthocyanins 249–447 mg/100 g fruits, proanthocyanidins 2.46–3.74 mg PB2E/100 g fruits (HPLC/spectrophotometry)	in vitro enzyme inhibition; control: acarbose IC_50_ = 130 ± 20 μg/mL	α-glucosidase inhibition: 80% EtOH IC_50_ = 0.70–0.88 μg/mL; acidified MeOH IC_50_ = 0.030–0.049 μg/mL (depending on the cultivars); anthocyanins responsible for observed effects (based on previous studies and extract composition)	[13]
	lyophilised wine samples prepared with or without additional sugar and enzyme; detected compounds (HPLC-MS): catechin; epicatechin; protocatechuic, gallic, chlorogenic, caffeic, *p*-coumaric, and ellagic acids	in vitro enzyme inhibition; control: acarbose IC_50_ = 77.8 ± 5.7 μg/mL	α-glucosidase inhibition: IC_50_ = 49–50 μg/mL (without additional sugar); IC_50_ = 28–30 μg/mL (with additional sugar); chlorogenic and caffeic acids’ contributions to the IC_50_ values: 16.4–19.5% and 4.78–6.15%, respectively (based on inhibitory curves of pure compounds and statistical analysis)	[14]
	MeOH and water dry extracts	in vitro enzyme inhibition; control: acarbose IC_50_ = 1.31 ± 0.12 μg/mL	α-amylase inhibition: MeOH (2.5 mg of extract/mL) inhibition = 58.6 ± 1.9%; water extract (2.5 mg of extract/mL) inhibition = 49.7 ± 3.8%	[56]
	60% EtOH and water extracts	in vitro enzyme inhibition; control: acarbose IC_50_ = 2.4 ± 0.4 μg/mL	α-amylase inhibition: water extract IC_50_ = 2632 ± 208.5 μg/mL, 60% EtOH IC_50_ = 1130 ± 91.19 μg/mL	[57]
	water, MeOH, and acetic acid extracts; composition (mg/100 mg) of acetic acid extract (HPLC-MS): neochlorogenic acid 4.22 mg, chlorogenic acid 1.26 mg, cyanidin monoglycosides 8.94 mg	in vitro enzyme inhibition; control: -	α-amylase inhibition: MeOH IC_50_ = 10.31 ± 0.04 mg/mL; water 13.55 ± 0.04 mg/mL; acetic acid 14.85 ± 0.06 mg/mL; the activity of pure compounds: the strongest for chlorogenic acid (IC_50_ = 0.57 ± 0.16 mg/mL) and cyanidin-3-glucoside (IC_50_ = 1.74 ± 0.04 mg/mL)	[58]
*Aronia prunifolia* (Marshall) Rehder	80% EtOH and acidified MeOH extracts; polyphenols 175 mg GAE/g of EtOH extract (2996 mg GAE/100 g of fresh fruits), anthocyanins 737 mg/100 g fruits, proanthocyanidins 4.79 mg PB2E/100 g fruits (HPLC/spectrophotometry)	in vitro enzyme inhibition; control: acarbose IC_50_ = 130 ± 20 μg/mL	α-glucosidase inhibition: 80% EtOH IC_50_ = 0.88 ± 0.08 μg/mL; acidified MeOH IC_50_ = 0.030 ± 0.005 μg/mL; anthocyanins responsible for observed effects (based on previous studies and extract composition)	[13]
*Chaenomeles japonica* (Thunb.) Lindl. ex Spach	60% EtOH and water extracts before in vitro digestion, water extract after digestion; detected compounds (HPLC-MS): epicatechin, procyanidin B2, procyanidin oligomers, quercetin-*O*-hexoside, epigallocatechin-3-gallate, catechin/epicatechin gallate	in vitro enzyme inhibition; control: acarbose IC_50_ = 2.4 ± 0.4 μg/mL	α-amylase inhibition (before digestion): water extract IC_50_ = 53.61 ± 5.074 μg/mL, 60% EtOH IC_50_ = 48.69 ± 4.993 μg/mL; α-amylase inhibition (after digestion): 55.41–58.48% inhibition at 50 μg/mL after gastric condition and about 50% after intestinal condition; catechin/epicatechin derivatives responsible for observed effects (based on literature studies and extract composition)	[57]
	70% acetone extract; detected compounds (HPLC): epigallocatechin (23.17 mg/g), (+) catechin, procyanidin B1, procyanidin C1, (−) epicatechin, epigallocatechin gallate, epicatechin gallate, hydroxybenzoic acids, hydroxycinnamic acids, and flavonols	in vitro enzyme inhibition and cellular studies (on βTC3 pancreatic β-cells); control: sodium orthovanadate (for PTP1B)	α-amylase inhibition: IC_50_ = 1.68–2.41 mg/mL depending on assay (better than, i.a., mulberry fruits); α-glucosidase inhibition: unable to detect; PTP1B inhibition: IC_50_ = 1.22 mg/mL (better than, i.a., mulberry fruits); cytoprotection of βTC3 pancreatic β-cells: highly positive effects on cell viability and proliferation; flavan-3-ols responsible for observed effects (based on literature studies and extract composition)	[36]
	70% acetone extract; polyphenols 489.85 mg/g of dry preparation, including (+)-catechin, (−)-epicatechin, epigallocatechingallate, procyanidins B1 and C1, hydroxybenzoic acids, and flavonols (HPLC)	cellular studies on the human hepatoma cell line HepG2; control: -	↑ level of *p*-AMPK compered to cells under normal or hyperglycaemic conditions; genes’ expression (5 mg/mL extract vs. cells under normal conditions): ↑ GLUT-4, IRS-2; ↓ PEPCK, PGC-1α, FOXO1, PTP1B; unchanged GLUT-2, G6Pase, GYS2, IRS-1, GSK-3α; genes’ expression (hyperglycaemic conditions): ↑ GLUT-2; ↓ PEPCK; unchanged GLUT-4, G6Pase, GYS2, IRS-1, IRS-2, GSK-3α, PTP1B, PGC-1α, FOXO1; glycogen content: ↑ at 5 mg/mL to a higher level than with metformin (at 5 mM); glucose production: ↑ at 5 mg/mL (weaker activity then metformin); glucose uptake: ↑ at 5 mg/mL to a higher level than with metformin (at 5 mM)	[29]
*Chaenomeles speciosa* (Sweet) Nakai	20, 40, 60, 80, and 100% MeOH; water; 20, 40, 60, 80, and 100% EtOH extracts; and crude polysaccharide fraction; polyphenols 2–318 mg GAE/g (HPLC: catechin; epicatechin; chlorogenic, gallic, caffeic, protocatechuic, *p*-coumaric, syringic, and vanillic acids), triterpenes 23–62 mg OAE/g (oleanolic and ursolic acids), polysaccharides 8–64 mg glucose/g	in vitro enzyme inhibition; control: -	α-glucosidase-inhibitory activity: IC_50_ about 0.2–6.2 mg/mL, the highest activity for 60% EtOH, 20% EtOH, and 60% MeOH extracts; α-glucosidase-inhibitory activity (pure compounds detected in the samples: about 10–100% inhibition at 0.5 mg/mL, the highest activity for the polysaccharide fraction and oleanolic acid); polysaccharides, oleanolic acid, and different polyphenols responsible for observed effects (based on activity studies of pure compounds and PCA analysis)	[59]
	water extract before and after gastric and/or intestinal digestion; polyphenols 7.87–12.7 mg GAE/g (HPLC: phenolic acids, catechin, epicatechin), triterpenes 39–48 mg OAE/g (ursolic and oleanolic acids), anthocyanins 0.6–1.6 mg CyE/g	in vitro enzyme inhibition and influence on starch digestion; control: -	α-glucosidase inhibition: IC_50_ about 0.35–0.7 mg/mL, with the lowest activity after intestinal digestion; effects on the glucose release during in vitro digestion (from corn starch): 98% inhibition, higher then observed for pure native compounds (25–65% inhibition)	[60]
	80% EtOH extracts (different origin of fruits); polyphenols about 19–29 mg GAE/g of plant material, flavonoids about 22–47 mg QE/g, polysaccharides about 20–26 mg glucose/g	in vitro enzyme inhibition; control: quercetin	α-glucosidase inhibition: IC_50_ about 60–210 mg QE/g of plant material (depending on fruit origin); flavonoids and polysaccharides responsible for observed activity (based on correlation studies)	[61]
*Chaenomeles speciosa* (Sweet) Nakai, *Chaenomeles sinensis* (Thouin) Koehne	60% MeOH extracts from peels, flesh, and endocarps of two species (*C. speciosa*, CSP; *C. sinensis*, CSS); polyphenols 356–405 (CSP) and 345–590 (CSS) mg GAE/g, triterpenes 29–43 (CSP) and 16–40 (CSS) mg OAE/g, detected compounds (HPLC-MS): five phenolic acids, two triterpenes, and three flavonoids	in vitro enzyme inhibition; control: -	α-glucosidase inhibition (*C. speciosa*): IC_50_ = 1.32–2.66 mg/mL, with the highest activity for the extract from flesh; α-glucosidase inhibition (*C. sinensis*): IC_50_ = 0.44–2.28 mg/mL, with the highest activity for the extract from the peel; α-glucosidase inhibition ratio after in vitro digestion: ↓ about 2–20-fold depending on the sample ferulic acid and triterpenes responsible for observed effects (based on correlation studies)	[62]
	60% MeOH extracts from peels and flesh of *C. speciosa* (CSP, 12 varieties) and *C. sinensis* (CSS, 1 variety); polyphenols about 100–270 (CSP, flesh), 170–360 (CSP, peel), 200 (CSS, flesh), and 350 mg GAE/g (CSS, peel); detected compounds (HPLC-MS): catechin; epicatechin; rutin; hyperoside; myricetin; quercetin; kaempferol; chlorogenic, gallic, caffeic, protocatechuic, syringic, oleanolic, and ursolic acids	in vitro enzyme inhibition; control: -	α-glucosidase inhibition (*C. speciosa*): IC_50_ about 0.06–0.35 mg/mL (peel extracts) and about 0.04–0.42 mg/mL (flesh extracts), with the highest activity for the extracts from flesh or peel, depending on the variety; α-glucosidase inhibition (*C. sinensis*): IC_50_ about 0.05 mg/mL (peel extract) and about 0.07 mg/mL (flesh extract); α-glucosidase inhibition of pure compounds detected in the samples: IC_50_ about 0.05–1.8 mg/mL (with the highest activity for hyperoside, quercetin, mirycetin, catechin, epicatechin, protocatechuic acid, chlorogenic acid, and oleanolic acid)	[63]
*Chaenomeles japonica* (Thunb.) Lindl. ex Spach, *Chaenomeles speciosa* (Sweet) Nakai, *Chaenomeles × superba* (Frahm) Rehder.	80% MeOH (1% HCl) extracts of *C. japonica* (5 cultivars), *C. speciosa* (3 cultivars), and *C. superba* (11 cultivars); polyphenols 56–170 mg/g, (HPLC-MS: (+)-catechin; (−)-epicatechin; procyanidins B2, B3, and C1, procyanidin oligomers and polymers; chlorogenic acids); contents of pectins, sugars, and organic acids also determined	in vitro enzyme inhibition; control: -	α-amylase inhibition: 16.11–17.45 mg/mL (*C. japonica*), 16.88–18.48 mg/mL (*C. speciosa*), 13.88–18.25 mg/mL (*C. superba*); α-glucosidase inhibition: 6.09–15.19 mg/mL (*C. japonica*), 5.74–12.48 mg/mL (*C. speciosa*), 2.67–8.54 mg/mL (*C. superba*); sugars, L-ascorbic acid, and flavan-3-ols responsible for α-amylase- and α-glucosidase-inhibitory activity (based on hierarchical clustering analysis)	[64]
*Cotoneaster bullatus* Bois, *Cotoneaster zabelii* C.K.Schneid., *Cotoneaster integerrimus* Medik.	70% MeOH extract; polyphenols 62.13–81.26 mg GAE/g (HPLC: chlorogenic acids, (−)-epicatechin, procyanidins B2 and C1, quercetin 3-(2”-xylosyl)galactoside, rutin, hyperoside, isoquercitrin, quercitrin)	in vitro enzyme inhibition and effects on AGE formation; Control: acarbose (IC_50_ = 169.52 and 5.78 µg/mL for α-glucosidase and α-amylase, respectively), aminoguanidin (IC_50_ = 71.09 µg/mL for AGEs)	α-glucosidase inhibition: IC_50_ = 48.89, 57.73, 80.12 µg/mL for *C. integerrimus, C. zabelii,* and *C. bullatus* (IC_50_ = 232–416 µg/mL for (−)-epicatechin, hyperoside, quercetin 3-(2”-xylosyl)galactoside, and procyanidin B2)*;* α-amylase inhibition: IC_50_ = 941–1083 µg/mL; inhibition of AGE formation: IC_50_ = 106.36, 118.94, 166.62 µg/mL for *C. integerrimus, C. zabelii,* and *C. bullatus* (IC_50_ = 2.20–15.78 µg/mL for procyanidin B2, (−)-epicatechin, hyperoside, and quercetin 3-(2”-xylosyl)galactoside)	[15]
*Crataegus azarolus var. aronia* L.	water extract	in vitro enzymatic starch digestion and glucose movement; control: acarbose 0.1 mg/mL (97.6% reduction in starch digestion); guar gum 50 mg/mL (glucose movement assay, iAUC ↓ by 30.8%)	enzymatic starch digestion: dose-dependent ↓ in glucose level, significant effect at 0.5–10 mg/mL (22.0–70.7% reduction, IC_50_ = 3.5 mg/mL); glucose movement (postprandial glucose level in vitro): lack of effects	[65]
*Crataegus laevigata* (Poir.) DC.	MeOH and water extracts	in vitro enzyme inhibition; control: acarbose IC_50_ = 1.31 ± 0.12 μg/mL	α-amylase inhibition: MeOH (2.5 mg of extract/mL), 35.4 ± 4.7% inhibition; water extract (2.5 mg of extract/mL), 41.1 ± 15.3% inhibition	[56]
	80% EtOH extract and fractions (EtOAc, *n*-butanol); polyphenols 136.54–697.23 mg GAE/g, flavonoids 21.46–154.51 mg QE/g, flavonols 19.21–68.83 mg QE/g	in vitro enzyme inhibition; control: acarbose IC_50_ = 275.43 ± 1.59 μg/mL	α-glucosidase inhibition: IC_50_ = 16.12–30.80 μg/mL, with the highest activity for the *n*-butanol fraction; caffeic acid, epicatechin, naringenin and quercetin responsible for observed effects (based on literature studies and extract composition)	[16]
*Crataegus microphylla* K. Koch	EtOH, acidified (0.5% HCl) EtOH, 50% EtOH, MeOH, acidified MeOH, 50% MeOH, water, acidified water extracts; polyphenols 5.00–57.28 mg GAE/g	in vitro enzyme inhibition; control: acarbose IC_50_ = 31.92 ± 0.08 μg/mL	α-glucosidase inhibition: IC_50_ = 250.94–731.81 μg/mL, with the highest activity for the acidified MeOH extract	[66]
*Crataegus pinnatifida* Bunge	MeOH extract and fractions (methylene chloride, EtOAc, *n*-butanol, water fractions); isolated compounds: hyperoside, chlorogenic acid, 3-epicorosolic acid, ursolic acid, oleanolic acid, β-sitosterol, β-sitosterol glucoside	in vitro enzyme inhibition and effects on the formation of AGEs; control: acarbose IC_50_ = 81.65 ± 4.07 μg/mL (α-glucosidase), ursolic acid IC_50_ = 1.00 ± 0.09 μg/mL (PTP1B), quercetin IC_50_ = 0.75 ± 0.07 μg/mL (ALR), aminoguanidine IC_50_ = 127.06 ± 7.10 μg/mL (AGEs)	α-glucosidase inhibition: IC_50_ = 22.70–122.11 μg/mL; PTP1B inhibition: IC_50_ = 1.41–18.75 μg/mL; Rat lens ALR inhibition: IC_50_ = 9.09–160.54 μg/mL; inhibition of AGEs formation: IC_50_ = 65.83–88.90 μg/mL; the highest activity observed for EtOAc (α-glucosidase, PTP1B, ALR inhibition) or MeOH extract (AGE formation); inhibitory potential of 3-epicorosolic acid IC_50_ = 4.08 and 30.18 μg/mL (PTP1B and α-glucosidase tests); different compounds responsible for observed effects (based on extract composition and literature studies)	[17]
	MeOH extract; polyphenols 101.56 mg tannic acid equivalents/g, flavonoids 44.52 mg CE/g	in vitro enzyme inhibition; control: acarbose IC_50_ = 2.15 ± 0.86 μg/mL	α-glucosidase inhibition: IC_50_ = 766.22 ± 8.14 μg/mL	[67]
	water extract before and after gastric and/or intestinal digestion; polyphenols 3.07–11.7 mg GAE/g (HPLC: five phenolic acids, catechin, epicatechin), triterpenes 28.4–47.4 mg OAE/g (ursolic and oleanolic acids), anthocyanins 0.77–1.8 mg CyE/g	in vitro enzyme inhibition and influence on starch digestion; control: -	α-glucosidase inhibition: IC_50_ about 0.3–0.75 mg/mL, with the lowest activity after intestinal digestion; effects on glucose release during in vitro digestion (from corn starch): 83% inhibition, higher then observed for pure native compounds (25–65% inhibition)	[60]
	MeOH, EtOH, EtOAc, acetone, dichloromethane, chloroform, *n*-hexane extracts; polyphenols 12.1–63.5 mg GAE/g (HPLC: chlorogenic acid, hyperoside, epicatechin, procyanidin B2)	in vitro enzyme inhibition and molecular docking studies; control: acarbose IC_50_ = 317.80 ± 16.36 μg/mL	α-glucosidase inhibition: IC_50_ = 42.35–207.46 μg/mL, with the highest activity for acetone, MeOH, and EtOH extracts (IC_50_ = 42.35–58.69 μg/mL); activity of isolated compounds: IC_50_ = 34.98–170.37 μg/mL, with the highest activity for hyperoside (also high affinity for enzyme/molecular docking studies)	[68]
*Cydonia oblonga* Mill.	70% EtOH macerate	in vitro enzyme inhibition; control: acarbose IC_50_ = 275.98 ± 1.57 μg/mL	α-glucosidase inhibition: IC_50_ = 326.48 ± 18.56 μg/mL	[69]
	70% EtOH extract from pulp fruit callus; polyphenols 10.98 mg/100 g (pulp) and 91.58 mg/100 g (callus), mainly chlorogenic acid, 5-*p*-coumaroylquinic acid, neochlorogenic acid, and (−)-epicatechin (LC-MS, GC-MS)	in vitro enzyme inhibition; control: acarbose about 75% and 50% inhibition at 32 μg (α-amylase and α-glucosidase, respectively)	α-amylase inhibition: about 25% inhibition at 250–1000 μg; α-glucosidase inhibition: about 40% inhibition at 250 μg and about 75% inhibition at 500 μg	[70]
*Cydonia oblonga* Mill.; *Malus domestica* (Suckow) Borkh.	fruit puree; flavan-3-ols 355.3 and 34.1 mg/kg (*Cydonia* and *Malus*, respectively), flavonols 1269 and 245.1 mg/kg, hydroxycinnamic acid derivatives 306.6 and 140.6 mg/kg, anthocyanins 62.2 and 67.6 mg/kg (HPLC)	in vitro enzyme inhibition; control: acarbose	α-amylase inhibition: IC_50_ = 164 and 161 mg/mL (*Cydonia* and *Malus*, respectively); α-glucosidase inhibition: IC_50_ = 177 and 166 mg/mL (*Cydonia* and *Malus*, respectively)	[71]
*Malus domestica* (Suckow) Borkh.	80% EtOH extract; 15 phenolics detected (HPLC), with phlorizin and chlorogenic acid as the dominant compounds	cellular studies on the human hepatoma cell line HepG2 with insulin resistance induced by high glucose levels (tested) or normal cells (control)	glucose uptake and glycogen content: ↑ in comparison to insulin-resistant cells (phlorizin and chlorogenic acid activity: comparable); protein levels: ↑ *p*-IRS2/IRS2, ↑ *p*-AKT/AKT, ↑ *p*-GSK3β/GSK3β, ↑ *p*-FOXO1/FOXO1, *p*-IRS1/IRS1 unchanged (insulin-resistant cells); phlorizin activity (10 μg/mL): comparable; chlorogenic acid: not active	[37]
	Phytonutriance^®^ polyphenolic extract Appl’In™; polyphenols min. 80%, phlorizin > 5%	cellular studies on Ishikawa Var 1 endometrial cells; control: genistein; about 30% inhibition at 2.7 μg/mL	glucose uptake: > 60% inhibition at 50 μg/mL (phlorizin equivalent 2.5 μg/mL); phlorizin responsible for observed effects (based on literature studies and extract composition)	[72]
	standardised commercial extract (Appl’In by DIANA Food SAS); polyphenols 67%, including 40% flavonoid monomers and phenolic acids (HPLC: flavan-3-ols, dihydrochalcones, flavonols, hydroxycinnamic acids)	cellular studies on Caco-2 cells and *Xenopus* oocytes injected to express SGLT1; control: cells without extract	total and sodium-independent (GLUT-mediated) glucose uptake (Caco-2 cells): 51% and 46% ↓ at 0.3 mg polyphenols/mL (corresponding to the physiological dose that may be reached after consumption of 600 mg of polyphenols (900 mg of apple extract) in the human study); glucose uptake in oocytes (SGLT1 mediate): ↓ at 0.125–4.0 mg apple polyphenols/mL (dose-independent), for phloretin and phlorizin 59% and 85% ↓ at 0.5 mM, respectively; different compounds responsible for observed effects (based on literature studies and extract composition)	[32]
	standardised commercial extract (Appl’In, Diana Naturals, France); polyphenols min. 80%, phlorizin > 5%	cellular studies on Caco-2 cells; control: dapagliflozin IC_50_ for SGLT1 inhibition ~0.5 µM = 0.2 mg/L	glucose uptake: 90% ↓ of SGLT1 at 0.2 mg/mL; ↓ GLUT-2 IC_50_ = 0.45 mg/mL (in comparison: phlorizin IC_50_ for SGLT1 = 0.13 mg/L, phloretin IC_50_ for GLUT-2 = 22 mg/L)	[34]
	standardised commercial extract (Appl’In, Diana Naturals, France); polyphenols min. 80%, phlorizin > 5%; 14 compounds detected (HPLC), including quercetin glycosides, dihydrochalcones, phenolic acids, and procyanidin oligomers	cellular studies on Caco-2 cells (TC7 subclone); control: cells without extract	glucose uptake: ↓ at 1.12 µg/mL (IC_50_ = 1.19 ± 0.35 mg/mL.); different compounds responsible for observed effects (based on literature studies and extract composition)	[73]
	80% MeOH extract (+0.1% formic acid); polyphenols 3.61 mg/g of fresh fruits, including flavanols, flavones, flavonols, dihydrochalcones, hydroxycinnamic acids, and anthocyanins (18 compounds, HPLC)	cellular studies on Caco-2 cells; control: cells with high glucose (HG) content and normal cells (NC)	formation of AGEs: ↓ compared to HG, the level at 0.8 mmol/L comparable to NC; glycolaldehyde-modified proteins: ↓ compared to HG, the levels at 0.4 and 0.8 mmol/L were comparable to NC; glyoxalase I activity: ↑ in the HG cells; at similar levels in the NC and tested cells (0.4–0.8 mmol/L extract); glyoxalase II activity: unchanged	[74]
	MeOH extract from apple juice; 10 phenolics detected (chlorogenic acid 0.33 mM, procyanidins dimer B type 0.05 mM, (−)-epicatechin 0.06 mM, *p*-coumaroylquinic acid 0.085 mM, phloretin glycosides 0.09 mM, quercetin and kaempferol glycosides 0.09 mM) (HPLC-MS)	cellular studies on Caco-2 cells; control: cells without extract or phenolics	glucose transport: ↓, with greater inhibition under sodium-free conditions (apical GLUT-2, IC_50_ = 136 mg) than under sodium-dependent conditions (SGLT1 and GLUT-2, IC_50_ = 300 mg); contribution of phenolics to observed effect: quercetin-3-*O*-rhamnoside 26% (IC_50_ = 31 μM), phlorizin 52% (IC_50_ = 146 μM), chlorogenic acid 12% (IC_50_ = 2570 μM)	[33]
	commercial extract (BioActive Food GmbH); polyphenols 44% (catechin equivalents), phlorizin 16%, quercetin 12.43%, chlorogenic acid 5.57% (HPLC/spectrophotometry)	cellular studies on *Xenopus* oocytes injected to express SGLT1; control: cells without extract	glucose uptake: ↓, extract IC_50_ = 2.0 ± 0.24 µg/mL; phlorizin IC_50_ = 0.46 ± 0.19 µM, quercetin IC_50_ = 0.62 ± 0.05 mM, chlorogenic acid not active	[75]
	juice: raw, after thermal or ultrasound pasteurisation and before or after in vitro digestion; polyphenols 5.33 mg/L (chlorogenic and *p*-coumaroylquinic acids, phlorizin and phloretin xyloqlucosides, epigallocatechin gallate, quercetin monoglycosides; HPLC-MS)	in vitro enzyme inhibition; control: acarbose IC_50_ = 0.09 μg/mL	α-glucosidase inhibition: IC_50_ = 6.24 mg/mL (raw sample), 0.92 mg/mL (sample after digestion); ↓ after thermal pasteurisation (not digested sample, 18.41 mg/mL) or ↑ (thermal pasteurisation, digested sample, 0.44 mg/mL); effects with acarbose: synergy at combinatory concentration of 2 mg/mL (i.e., up to 40% inhibition), antagonism at 2–9 mg/mL, and additive activity at >9 mg/mL; different compounds responsible for observed effects (based on literature studies and extract composition)	[25,76]
	DMSO extract; 5% quercetin, 30% phlorizin	in vitro receptor binding; control: rosiglitasone IC_50_ = 0.043 ± 0.004 µg/mL (binding affinity test)	binding affinity to PPARγ receptor: moderate (IC_50_ = 0.49 µg/mL); effects on rosiglitazone-mediated DRIP205/TRAP220 coactivator: strong antagonism (IC_50_ = 0.15 ± 0.03 µg/mL); phlorizin, phloretin, epicatechin and catechin responsible for observed effects (based on literature studies and apple composition)	[38]
	water and 12% EtOH extracts from peel and pulp, with or without different elicitor treatments; polyphenols 0.35–1.44 mg GAE/g of fresh fruits for peel, 0.05–0.32 mg GAE/g for pulp; detected compounds (HPLC): chlorogenic acid, *p*-coumaric acid, gallic acid, quercetin	in vitro enzyme inhibition; control: -	α-glucosidase inhibition: higher activity found for extracts from peel, as well as 12% EtOH extracts; the activity of extracts from peel (without elicitors) ↓ after fruit storage, and the activity of extracts from pulp ↑ and then ↓ depending on the storage time (0–3 months); α-amylase inhibition: higher activity found for extracts from pulp, as well as water extracts; the activity of extracts from peel (without elicitors) ↑ after storage, while the activity of extracts from pulp was similar after storage; chlorogenic acid and quercetin responsible for observed effects (based on literature studies and extract composition)	[77]
	12% EtOH and water extracts from peel and pulp of different cultivars; polyphenols 266–781 μg GAE/g fresh material for peel extracts and 30–143 μg GAE/g for pulp extracts; detected compounds (HPLC): catechin, chlorogenic, and *p*-coumaric acids; quercetin	in vitro enzyme inhibition; control: -	α-amylase inhibition: the highest activity for pulp (about 0–60% at 100 μL) or peel water extracts (about 0–55%); α-glucosidase inhibition: higher activity for pulp water extract (69–83% at 50 μL) than pulp 12% EtOH extract (50–70% at 50 μL); higher or similar activity for peel 12% EtOH extract (54–98% at 50 μL) than peel water extract (52–93% at 50 μL); polyphenols responsible for observed effects (based on correlation studies)	[78]
	acetone–ethanol extract (1:3), purified with 95% MeOH (polyphenols 390.8 μg CE/mg,) and fractionated with acetonitrile (II: polyphenols 61.3 μg CE/mg), EtOAc (III: polyphenols 459.3 μg CE/mg), MeOH (IV: polyphenols 620.6 μg CE/mg), and fraction I residue; 14 compounds detected (HPLC-MS)	in vitro enzyme inhibition; control: acarbose IC_50_ = 840 ± 100 μg/mL, quercetin 661 ± 7 μg/mL	α-glucosidase inhibition: IC_50_ = 19–67 μg/mL (purified 95% MeOH extract and fractions III-IV); chlorogenic acid isomers, flavan-3-ols monomers and oligomers, quercetin and phloretin glycosides responsible for observed effects (based on literature studies and extract composition)	[18]
	70% EtOH extract; polyphenols 534.39 mg GAE/g (HPLC: phlorizin; (−)-epigallocatechin; (−)-epicatechin; (+)-catechin; procyanidins B1 and B2; quercetin glycosides; chlorogenic, *p*-coumaroylquinic, and caffeic acids	in vitro enzyme inhibition and molecular docking studies; control: acarbose IC_50_ = 0.76 μg/mL	α-glucosidase inhibition: IC_50_ = 15 μg/mL; phlorizin, (−)-epicatechin, and tannins responsible for observed effects (based on activity study of pure compounds); inhibition mechanism: competitive or mixed type, potential conformational change of enzyme was suggested	[79]
	apple extract	effects on the formation of AGEs in plasma in vitro; control: -	concentration-dependent (at 100–2000 mmol) inhibition of AGE formation (up to fourfold ↓), depending on the glucose concentration (5.5–50 mmol) and time of study	[80]
	water extract from the juice;	in vitro enzyme inhibition; control: quercetin IC_50_ = 153.85 ± 5.38 μg/mL (ALR), 67.5 ± 0.8 μg/mL (SDH)	ALR inhibition: IC_50_ = 171.63 ± 5.42 μg/mL; SDH inhibition: IC_50_ = 56.52 ± 4.95 μg/mL	[41]
	juice (fermented and unfermented); compounds: 19 polyphenols (HPLC: mainly chlorogenic acid at about 95–145 mg/L, epicatechin 70–105 mg/L, procyanidin B2 70–95 mg/L), sugars, and organic acids	in vitro enzyme inhibition; control: acarbose, with about 60–80% inhibition at 0.025–0.1 mg/mL	α-glucosidase inhibition: about 43% inhibition at 0.1 mL/mL (fermented and unfermented samples); at 0.5 mL/mL, about 67% (unfermented) and 78% (fermented)	[81,82]
*Malus pumila* Mill.	80% MeOH extracts (0.5% formic acid) from the peel and flesh of different cultivars; polyphenols 25.12–281.73 mg/100 g, phlorizin 1.10–68.54 mg/100 g (peel > flesh), procyanidins 6.03–78.76 mg/100 g (flesh > peel) (HPLC)	cellular studies on HepG2 human hepatocellular liver carcinoma cells; control: cells without extracts	glucose uptake: ↑, with the highest activity for peel extract from the Red Delicious cultivar, with the highest total polyphenols and phlorizin content; phlorizin responsible for observed effect (based on correlation and literature studies)	[83]
	commercial extract (Exxentia^®^); polyphenols 57.5%, including phlorizin (9.9%), chlorogenic acid (15.8%), and quercetin (0.4%)	cellular studies on L6.C11 rat skeletal muscle myoblast cells (insulin sensitivity mechanisms) and ex vivo α-glucosidase inhibition on isolated rat intestinal mucosa; control: cells not treated with extract or rosiglitazone (Rosi, 10 μmol/L)	glucose uptake: maximal ↑ by 63.6%, EC_50_ = 4.2 ± 0.7 μg/mL; insulin-stimulated glucose uptake: increasing synergistic effects at 5–25 μg/mL of extract and 50 nM of insulin; GLUT-4 levels: not changed in total membranes, ↑ in a plasma membrane fraction (GLUT-4 translocation, at 25 μg/mL); ↑ *p*-Akt/Akt by about 50% (unchanged for Rosi); PPARγ level and PPARγ-mediated transcription ↑ (comparable to Rosi), ERK1/2 not affected (for extract at 25 μg/mL); α-glucosidase inhibition: IC_50_ 12.54–18.05 μg/mL (three models)	[30]
*Malus* Mill. sp.	fruit water extract	in vitro enzyme inhibition; control: acarbose	α-glucosidase inhibition: 8 mg acarbose equivalents/g	[84]
	apple juice; polyphenols 0.78 mg GAE/mL	effects on AGE formation in vitro control: -	weak inhibition (about 5%) at 10 µL of juice/mL compared to pomegranate juice (about 95% inhibition)	[85]
	MeOH extract from red or yellow apples; polyphenols about 4 mg GAE/g dry fruits	in vitro enzyme inhibition; control: -	α-amylase inhibition: not active; α-glucosidase inhibition: 49.5% (yellow apple) or 95.4% (red apple) inhibition at 10 mg/mL concentration;	[86]
*Malus* Mill. sp. (276 *Malus* species/cultivars including *Malus sieversii* and *Malus domestica*)	80% MeOH extract + 0.1% HCl + sonification; polyphenols 0.49–2.61 mg/g fresh fruits (HPLC-MS: phenolic acids, flavan-3-ols, proanthocyanidins, flavonols, dihydrochalcones)	in vitro enzyme inhibition and effects on AGE formation; control: acarbose (IC_50_ = 0.21 mg/mL and 0.58 µg/mL for α-glucosidase and α-amylase), sitagliptin (IC_50_ = 0.044 µg/mL for DPP IV), aminoguanidin (IC_50_ = 24 µg/mL for AGEs)	α-glucosidase inhibition: IC_50_ = 7.1–256 mg/mL (0.01 mg/mL for phlorizin, 0.028–0.073 mg/mL for chlorogenic acid, epicatechin, procyanidin B2, and quercetin 3-*O*-galactoside); α-amylase inhibition: IC_50_ = 5.3–21.5 mg/mL for 10 cultivars with the highest total polyphenols (485 and 749 µg/mL for quercetin 3-*O*-galactoside and epicatechin); DPP IV inhibition: 10.3 mg/mL to inactive for 10 selected cultivars (75 and 90 µg/mL for chlorogenic acid and quercetin 3-*O*-galactoside); inhibition of AGE formation: IC_50_ = 5.2 mg/mL to inactive for 10 selected cultivars (17.1 and 23 µg/mL for quercetin 3-*O*-galactoside and epicatechin)	[42]
*Mespilus germanica* L.	acidified 80% EtOH extract, along with its water and 80% MeOH fractions; polyphenols 32.05–74.93 mg/g (HPLC: phenolic acids, procyanidin B2, catechin, and epicatechin)	in vitro enzyme inhibition; control: acarbose 100% inhibition at 50 mg/mL	α-amylase inhibition: about 47%, 27%, and 52% inhibition at 5 mg/mL for EtOH, MeOH, and water fractions, respectively; phenolic acids (e.g., gallic, chlorogenic, and ferulic acids) responsible for observed effects (based on literature studies and extract composition)	[87]
	EtOH and water extracts; polyphenols 6.93 mg GAE/g dry fruits	in vitro enzyme inhibition; control: -	α-amylase inhibition: not active; α-glucosidase inhibition: 71.5% at 20 mg/mL for EtOH, 44.9% for water extract (comparable to onion extract)	[88]
	EtOH extract; polyphenols 16.5 mg GAE/g, flavonoids 1.99 mg QE/g	in vitro enzyme inhibition; control: acarbose, with about 85% inhibition at 10 μg/mL (α-amylase), and about 85% inhibition at 25 μg/mL (α-glucosidase)	α-amylase inhibition: about 35% at 10 and 100 μg/mL; α-glucosidase inhibition: about 99% at 25 μg/mL	[89]
*Pyracantha fortuneana* (Maxim.) H.L.Li	proanthocyanidin fraction from 70% acetone extract; compounds (HPLC): epicatechin, catechin, A-type and B-type procyanidins, procyanidin glucosides	in vitro enzyme inhibition and molecular docking studies; control: acarbose IC_50_ = 307 ± 1 μg/mL	α-glucosidase inhibition: IC_50_ = 0.15 ± 0.01 μg/mL; reversible, non-competitive inhibition; alteration of the catalytic configuration of the enzyme’s active site; procyanidins responsible for observed effects (based on extract composition and molecular docking studies)	[20]
	50–90% MeOH, EtOH and acetone extracts; polyphenols 9.67–17.33 mg GAE/g, 25 compounds detected (HPLC-MS: flavonoids and phenolic acids), flavonoids 0.34–1.03 mg QE/g, polysaccharides 72.87–103.65 mg glucose/g	in vitro enzyme inhibition; control: acarbose IC_50_ = 1071 ± 29 μg/mL	α-glucosidase inhibition: IC_50_ = 350–1870 μg/mL, with the highest activity observed for 50% and 70% acetone extracts; polyphenols responsible for observed effects (correlation studies)	[19]
*Pyrus bretschneideri* Rehder	60% MeOH from peel and pulp; polyphenols about 2.9 and 8.1 mg GAE/g in pulp and peel, respectively (HPLC-MS: catechin; epicatechin; rutin; chlorogenic, *p*-coumaric, vanillic, gallic, and ferulic acids), flavonoids about 1.5 and 6.3 mg RE/g, terpenes about 0.9 and 4.3 mg OAE/g (oleanolic and ursolic acids)	in vitro enzyme inhibition; control: compounds detected in the tested extracts, 2–89% inhibition at 40 μg/mL	α-glucosidase inhibition: IC_50_ 190 μg/mL for peel and 1220 μg/mL for pulp extract; activity of model compounds: the highest activity found for ferulic acid, rutin, and vanillic acid (about 80–89% inhibition at 40 μg/mL)	[90]
*Pyrus communis* L.	MeOH extracts from peel, flesh, or peel + flesh of different *Pyrus* cultivars	in vitro enzyme inhibition; control: acarbose 79.75 ± 1.86% inhibition at 0.5 mg/mL (α-amylase), 70.16 ± 1.60% inhibition at 0.5 mg/mL (α-glucosidase)	α-amylase inhibition: 1.20–18.49% for peel at 6 mg/mL, flesh or flesh + peel not active; α-glucosidase inhibition: highly dependent on the cultivar, with the highest activity for “Takiša”, i.e., 76.50–99.64% inhibition at 0.5 mg/mL; for wild *Pyrus communis*, 13.59% inhibition at 0.5 mg/mL and 63.36% at 1.0 mg/mL (peel)	[91]
	juice before or after fermentation; polyphenols about 0.25–0.6 mg GAE/g fresh fruits (HPLC: catechin; rutin; chlorogenic, *p*-coumaric, protocatechuic, benzoic, and gallic acids)	in vitro enzyme inhibition; control: -	α-amylase inhibition: ↑ after fermentation; α-glucosidase inhibition: ↑, ↑ or unchanged after fermentation (depending on the length, pH, and cultivar)	[92]
	juice from different cultivars before or after fermentation; proteins 3.8–7.8 mg/mL, phenolics about 0.4 mg/mL (HPLC: epicatechin, *p*-coumaric and caffeic acids, quercetin derivatives)	in vitro enzyme inhibition; control: -	α-amylase inhibition: not active; α-glucosidase inhibition: ↑ or ↑ after fermentation (depending on the length, bacteria, and pH), about 5–80% inhibition at 10–50 μL	[93]
	12% EtOH and water extracts from peel and pulp of different cultivars; polyphenols 270–1300 μg GAE/g fresh material for peel and 27–150 μg GAE/g for pulp (HPLC: chlorogenic, caffeic, protocatechuic, p-coumaric, and gallic acids; catechin; quercetin derivatives)	in vitro enzyme inhibition; control: -	α-amylase inhibition: the highest activity for pulp water extract (about 20–50% at 100 μL); α-glucosidase inhibition: depending on the study and cultivar (about 10–60% at 10 μL); polyphenols responsible for observed effects (based on correlation studies or extract composition)	[94,95]
*Pyrus pashia* Buch.-Ham ex D. Don	EtOAc fraction of 70% EtOH extract before or after NaOH hydrolysis; tannins 780 mg CE/100 g of fresh fruits, sugars 15.93 g/100 g	in vitro enzyme inhibition; control: acarbose IC_50_ = 55 and 440 μg/mL (α-amylase and α-glucosidase)	α-amylase inhibition: IC_50_ = 72–100 μg/mL; α-glucosidase inhibition: IC_50_ = 85–330 μg/mL	[21]
*Pyrus pyrifolia* (Burm.f.) Nakai	80% EtOH + 70% acetone extract from two cultivars fractionated on Sephadex; polyphenols 20.9–28.5 mg CE/g	in vitro enzyme inhibition; control: -	α-glucosidase inhibition: IC_50_ = 21.3–66.4 μg/mL; oligomeric and polymeric polyphenols responsible for observed effects (based on activity testing of phenolic fractions)	[96]
	water extract	in vitro enzyme activity; control: acarbose about 10–40% inhibition at 0.05–1 mg/mL	GK activity: ↑ at 5 and 10 mg/mL; α-glucosidase inhibition: about 10–30% at 0.25–1 mg/mL	[39]
	50% EtOH extract from pear pomace;	cellular studies on 3T3-L1 mouse cells; control: cells not treated with extract (glucose uptake), rosiglitazone 1 μM (Rosi, for protein expression)	glucose uptake: ↑ at 100 and 250 μg/mL; protein expression: ↑ *p*-IRS-1 (Tyr632) and *p*-Akt at 100 μg/mL (better than Rosi), ↑ GLUT-4 (comparable to Rosi)	[31]
*Pyrus pyrifolia* (Burm.f.) Nakai, *P. ussuriensis* Maxim. Ex Rupr., *P. betulifolia* Bunge, *P. bretschneideri* Rehder	juices before and after in vitro digestion; polyphenols about 0.18–0.4 mg/mL and 0.18–0.4 mg/mL, polysaccharides about 4.5–8 mg/mL and 4.5–12 mg/mL (before and after digestion, respectively)	in vitro enzyme inhibition; control: acarbose	α-amylase inhibition: about 1.2–1.6 mg acarbose/mL before digestion, 1.2–2.2 mg acarbose/mL after digestion; α-glucosidase inhibition: about 4.5–6.5 mg acarbose/mL before digestion, 5–12.5 mg acarbose/mL after digestion; polyphenols and polysaccharides responsible for observed effects (based on correlation studies or extract composition)	[97]
*Sorbus aucuparia* L.	80% acetonitrile extract (whole) and fractions (Sephadex); compounds in the whole extract: hydroxycinnamic acids (chlorogenic acids), flavonols, proanthocyanidins, anthocyanins (LC-MS)	in vitro enzyme inhibition; control: acarbose IC_50_ = 0.08 μg/mL	α-amylase inhibition: whole-extract IC_50_ = 4.5 μg GAE/mL; the activity of the proanthocyanidin-rich fraction was comparable to that of the whole extract; synergy with acarbose; proanthocyanidins responsible for observed effects (based on activity testing of phenolic fractions)	[27]
	80% acetonitrile extract (whole) and fractions (Sephadex); compounds in the whole extract: 12 phenolics, including chlorogenic acid (65%), dicaffeoylquinic and coumaroylquinic acids, quercetin-3-*O*-glucoside, and caffeoyl glucose (LC-MS)	in vitro enzyme inhibition; control: acarbose IC_50_ = about 40 μg/mL	α-glucosidase inhibition: whole-extract IC_50_ = about 30 μg GAE/mL, synergy with acarbose, no synergy with other berries (blackcurrant); proanthocyanidin-rich fraction IC_50_ > 100 μg GAE/mL; chlorogenic acids responsible for observed effects (based on extract composition and literature studies)	[26]
	water extract; polyphenols 19.13 mg GAE/g, flavonoids 9.62 mg CE/g	in vitro enzyme inhibition; control: acarbose IC_50_ = 6 ± 0.2 μg/mL (α-amylase), IC_50_ = 86 ± 2.7 μg/mL (α-glucosidase)	α-amylase inhibition: IC_50_ > 800 μg/mL; α-glucosidase inhibition: IC_50_ = 50 μg/mL; polyphenols responsible for observed effects (based on extract composition and literature studies)	[23]
	50% MeOH and 50% acetone extracts and fractions (Et_2_O, EtOAc, *n*-butanol, water); polyphenols 1.31–274.79 mg/g (51 compounds (HPLC-MS), including caffeic and ferulic acids pseudodepsides, flavonols, and proanthocyanidins)	effects on the formation of AGEs in vitro; control: aminoguanidine (AG)	inhibition of AGE formation: IC_50_ = 22.37–55.33 µmol AG/mg of extract (2–4-fold higher activity than AG); IC_50_ for chlorogenic acid, quercetin 3-*O*-β-sophoroside, and procyanidin B2: 152, 254, and 486 µmol AG/mg, respectively	[43]
	60% EtOH and water extracts	in vitro enzyme inhibition; control: acarbose IC_50_ = 2.4 ± 0.4 μg/mL	α-amylase inhibition: water extract IC_50_ = 1236 ± 177.0 μg/mL, 60% EtOH IC_50_ = 973.9 ± 61.60 μg/mL	[57]
*Sorbus decora* (Sarg.) CK Schneid.	80% EtOH extract	effects on the formation of AGEs in vitro; control: quercetin IC_50_ = 6.1 ± 1.8 μM (about 1.84 μg/mL)	inhibition of AGE formation: IC_50_ = 192.7 ± 37.1 μg/mL	[98]
*Sorbus domestica* L.	water extract	in vitro enzyme inhibition; control: acarbose IC_50_ = 120 ± 23 μg/mL (α-amylase), IC_50_ = 548 ± 21 μg/mL (α-glucosidase)	α-amylase inhibition: IC_50_ = 8768 μg/mL; α-glucosidase inhibition: IC_50_ = 417 μg/mL	[99]
	MeOH extract and fractions (dichloromethane, Et_2_O, EtOAc, butanol, water) of fruits at different stages of maturity; polyphenols 2.27–341 mg GAE/g (47 compounds (LC-MS), including flavonoids, benzoic and cinnamic acid derivatives, and biphenyls)	in vitro enzyme inhibition; control: sorbinil, 45% inhibition at 0.25 μM	ALR inhibition: 72–93% for Et_2_O and EtOAc fractions at 50 μg/mL, >50% inhibition for dichloromethane fraction, <40% inhibition for butanol and water fractions; flavonoids and hydroxycinnamoyl esters responsible for observed effects (based on extract composition and statistical analysis)	[44]
*Sorbus torminalis* (L.) Crantz	water extract; polyphenols 24.21 mg GAE/g, flavonoids 15.69 mg CE/g	in vitro enzyme inhibition; control: acarbose IC_50_ = 6 ± 0.2 μg/mL (α-amylase), IC_50_ = 86 ± 2.7 μg/mL (α-glucosidase)	α-amylase inhibition: IC_50_ = 307 μg/mL; α-glucosidase inhibition: IC_50_ = 27 μg/mL; polyphenols responsible for observed effects (based on extract composition and literature studies)	[23]
*Sorbus* species from subgenus *Aria* ** and *Sorbus* ***	80% acetone extracts and fractions (carbohydrates and phenolics, only from *S. norvegica*); detected compounds (NMR): chlorogenic and neochlorogenic acids, carbohydrates	in vitro enzyme inhibition; control: acarbose IC_50_ = 0.047 ± 0.006 μg/mL (α-amylase), IC_50_ = 742 ± 147 μg/mL (α-glucosidase),	α-amylase inhibition: IC_50_ = 2.5–12.3 μg/mL (*Aria*), the highest activity—*S. norvegica*; IC_50_ = 30.3–2540 μg/mL (*Sorbus*), the highest activity—*S. hybrid*, *S. aucuparia*, *S. meinichii*; α-glucosidase inhibition: IC_50_ = 0.9–3.7 μg/mL (*Aria*), the highest activity—*S. alnifolia, S. minima, S. norvegica*; IC_50_ = 4.6–300 μg/mL (*Sorbus*), the highest activity—*S. hybrida, S. aucuparia, S. meinichii;* carbohydrates and polyphenols contributed to the observed effects (based on correlation studies and activity testing of fractions from *S. norvegica*)	[22]
*Vauquelinia corymbose* Bonpl.	water extract; detected compounds (HPLC): prunasin, (−)-epicatechin, hyperoside, isoquercetin, quercitrin, quercetin-3-O-(6″-benzoyl)-β-galactoside, picein, methylarbutin	in vitro enzyme inhibition and molecular docking studies; control: acarbose IC_50_ = 500 μM (yeast α-glucosidase), IC_50_ = 100 μM (rat small intestinal α-glucosidase)	α-glucosidase inhibition: IC_50_ = 28.6 μg/mL (yeast α-glucosidase); the most active compound: quercetin-3-*O*-(6″-benzoyl)-β-galactoside (IC_50_ = 30 μM for yeast α-glucosidase and 430 μM for rat small-intestinal α-glucosidase; mixed-type inhibitor)	[24]

* Suggestions of what compounds were responsible for the observed effects were taken directly from the cited papers; for a critical discussion, see Section 3.4. ** Tested species from the subgenus Aria: *S. alnifolia* (Siebold & Zucc.) K.Koch., *S. folgneri* Rehder, *S. minima* Hedl., *S. norvegica* Hedl. *** Tested species from the subgenus Sorbus: *S. aucuparia* L., *S. commixta* Hedl., *S. decora* (Serg.) C.K. Schneid., *S. discolor* (Maxim.) Maxim., *S. hybrida* L., *S. meinichii* (Lindeb. ex Hartm.) Sennikov and Kurtto, *S. koehneana* C.K. Schneid., *S. vilmorinii* C.K. Schneid., *S. splendida* Hedl. ↑, increase; ↓, decrease; AGEs, advanced glycation end products; Akt, protein kinase B; ALR, aldose reductase; CE, catechin equivalents; CyE, cyanidin glucoside equivalents; DMSO, dimethyl sulfoxide; DRIP205/TRAP220, mediator of RNA polymerase II transcription subunit 1; ERK 1/2, extracellular signal-regulated kinases 1/2; Et2O, diethyl ether; EtOAc, ethyl acetate; EtOH, ethanol; FOXO1, forkhead box G1; G6Pase, glucose-6-phosphatase; G6PD, glucose-6-phosphate dehydrogenase; GAE, gallic acid equivalents; GK, glucokinase; GLUT-1, glucose transporter-1; GLUT-2, glucose transporter 2; GLUT-4, glucose transporter-4; GSK-3α, glycogen synthase kinase 3 alfa; GSK-3β, glycogen synthase kinase 3 beta; GYS2, glycogen synthase 2; HK1, hexokinase 1; HPLC, high-performance liquid chromatography; HPLC-MS, high-performance liquid chromatography coupled with mass spectrometry; iAUC, incremental area under the curve; IRS-1, insulin receptor substrate 1; IRS-2, insulin receptor substrate 2; LC-MS, liquid chromatography coupled with mass spectrometry; LPS, lipopolysaccharide; MeOH, methanol; OAE, oleanolic acid equivalents; p-Akt, phosphorylated Akt; p-AMPK, phosphorylated AMP-activated protein kinase; PB2E, procyanidin B2 equivalents; PEPCK, phosphoenolpyruvate carboxykinase; p-FOXO1, phosphorylated FOXO-1; PGC-1α, peroxisome proliferator-activated receptor gamma, coactivator 1 alpha; p-GSK-3β, phosphorylated GSK-3β; *p*-IRS-1, phosphorylated IRS-1; *p*-IRS-2, phosphorylated IRS-2; PCA, principal component analysis; PPARγ, peroxisome proliferator-activated receptor gamma; PTP1B, protein tyrosine phosphatase 1B; QE, quercetin equivalents; RE, rutin equivalents; SDH, sorbitol dehydrogenase; SGLT1, sodium–glucose transporter; SOCS3, suppressor of cytokine signalling 3.

### 3.2. In Vivo Animal Studies

The animal studies included different mouse, rat, or lamb models of diabetes, induced in vivo either chemically (e.g., streptozotocin, alloxan, dexamethasone) or by diet (for detailed information, see Table 2). The results suggested that fruits from *Amelanchier alnifolia, Aronia melanocarpa, Chaenomeles sinensis, Crataegus azarolus, Crataegus laevigata, Crataegus meyeri, Crataegus monogyna, Crataegus orientalis, Crataegus pinnatifida, Malus domestica, Malus pumila, Pyrus bretschneideri, Pyrus communis, Pyrus pyrifolia, Sorbus aucuparia,* and *Sorbus norvegica* can reduce blood/plasma glucose levels to varying degrees, depending on the animal model, plant species, and fruit preparation (e.g., berry powder, pomace, juices, extracts based on water, ethanol–water, or acetone–water, or their organic fractions). In the case of *Aronia melanocarpa* acidified 80% ethanol extract at 300 mg/kg/day [100], *Crataegus laevigata* 70% ethanol extract at 1200 mg/kg/day [101], *Crataegus pinnatifida* acidified 70% ethanol extract at 300 mg/kg/day [102], *Pyrus communis* 80% ethanol and ethyl acetate extracts at 200 mg/kg/day [103], and *Sorbus norvegica* 80% acetone extract at 900–1250 mg/kg/day [22], the blood glucose levels after extract supplementation were comparable with the effects of the reference anti-diabetic drugs, i.e., metformin (150–200 mg/kg/day), glipizide (10 mg/kg/day), glibenclamide (5 mg/kg/day), and acarbose (25 mg/kg/day). Moreover, the HOMA-IR index (homeostatic model assessment for insulin resistance, calculated based on glucose and insulin measurements) and QUICKI (quantitative insulin sensitivity check index) observed for *Amelanchier alnifolia* [104,105,106,107], *Cydonia oblonga* [108], *Malus domestica* [82,109], and *Pyrus pyrifolia* [31] suggested that the hypoglycaemic effects of fruits are driven by the improvement of insulin sensitivity rather than stimulation of insulin secretion. This was consistent with the blood/plasma insulin levels, which were reduced for all species and studies mentioned above, as well as for *Aronia melanocarpa* [100] and *Crataegus pinnatifida* [102,110]. Moreover, the ability of *Aronia melanocarpa* [100,111], *Crataegus monogyna* [112], *Crataegus pinnatifida* [102,110], and *Malus pumila* [113] fruits to ameliorate histological changes in pancreatic or liver cells, like hypertrophy or degradation, was also evidenced.

There were several mechanisms behind the activity observed in vivo that were tested for Maleae fruits (Table 2), including the following:

(1) The effects on intestinal absorption of glucose;
(2) The effects on skeletal, hepatic, or adipose transport of glucose;
(3) The changes in the expression of proteins involved in the insulin signalling pathway;
(4) The modulation of the activity of enzymes involved in glucose metabolism;
(5) The inhibition of glucose-derived protein damage.

(1) The impairment of intestinal glucose transporter (SGLT-1), which may decrease the absorption of sugars consumed with foods, was proven for *Malus domestica* [75]. Moreover, modification of the gut microbiota’s function was also reported to be involved in reducing intestinal sugar absorption. This effect was observed for fruits from *Amelanchier alnifolia*, which were able to, i.a., alter the α-diversity and β-diversity of gut microbiota and reduce the ratio of *Firmicutes*/*Bacteroidetes*, which was negatively correlated with carbohydrate digestion [104,105,106]. Finally, the inhibition of mucosal enzymes’ activity, i.e., sucrase and maltase (α-glucosidases that catalyse the hydrolysis of sucrose and maltose *O*-glycosidic bonds), was observed for the *Aronia melanocarpa* juice and extract and proposed as another mechanism of lowering the blood/plasma glucose levels by inhibiting the digestion and absorption of sugars [114,115].

(2) The intensification of hepatic (GLUT-2, GLUT-4), muscle (GLUT-4), and adipose (GLUT-1, GLUT-4) glucose transport was suggested for *Aronia melanocarpa* [35,100,116,117], *Crataegus pinnatifida* [102,118], and *Pyrus pyrifolia* [31] hydroalcoholic fruit extracts. Thus, these may increase tissue glucose uptake for further metabolism and lower the sugar level in the bloodstream.

(3) The homeostasis of glucose metabolism depends on several simultaneous ongoing processes under the control of insulin. The binding of insulin to its receptor leads to a cascade of reactions that promote glucose usage and storage by different tissues (liver, muscle, or adipose), e.g., the stimulation of glycogen synthesis (i.e., the transformation of glucose to glycogen) and glycolysis (i.e., the conversion of glucose to pyruvate and production of ATP energy). At the same time, de novo synthesis of glucose (gluconeogenesis) and glycogenolysis (metabolism of glycogen to glucose) are suppressed [50]. However, insulin secretion is only the beginning of the chain reactions, and these processes can be controlled at multiple stages (see Figure 2). As mentioned earlier, the results of the hypoglycaemic activity studies of Maleae fruits suggested that the reduction in blood/plasma glucose levels was driven by the improvement of insulin sensitivity rather than the stimulation of insulin secretion. Thus, the effects on the expression of different proteins in the insulin signalling pathway were tested for some species. Depending on the studies, the *Aronia melanocarpa* juices or extracts were able to increase the expression of *p*-PI3K (phosphorylated phosphoinositide 3-kinase), *p*-Akt (phosphorylated protein kinase B), GYS (glycogen synthase), and GLP-1 (glucagon-like peptide 1), as well as the ratios of *p*-IRS-1(2)/IRS-1(2) (phosphorylated insulin receptor substrate 1(2)/ insulin receptor substrate 1(2)) and *p*-GSK-3β/GSK-3β (phosphorylated glycogen synthase kinase 3 beta/ glycogen synthase kinase 3 beta); they were also documented to decrease the levels of PTEN (phosphatase and tensin homolog) and SOCS3 (suppressor of cytokine signalling 3), which may result in the enhancement of glycolysis and glycogen synthesis, as well as the inhibition of gluconeogenesis and glycogenolysis [100,115,116,117,119]. Similar effects were observed for *Crataegus pinnatifida*—by increasing *p*-Akt, *p*-AMPK (phosphorylated AMP-activated protein kinase), *p*-IRS-1, and *p*-PI3K, and decreasing PEPCK (phosphoenolpyruvate carboxykinase) levels [102,118]; various *Crataegus* spp.—by increasing the GPC-4 (glypican-4) level [120]; *Cydonia oblonga*—by increasing *p*-AMPK and decreasing PPARγ levels [108]; and *Malus pumila*—by increasing the *p*-Akt level [113]. On the other hand, the results of gene expression studies in *Amelanchier alnifolia* berry powder suggested the enhancement of opposing processes of glycolysis and gluconeogenesis, but the final effect on glucose metabolism was still positive, i.e., oral glucose tolerance test parameters were improved in comparison to diabetic controls [121]. In this case, the answer may be not in the levels of particular enzymes, but rather in their activity, which needs further investigation.

(4) Therefore, in addition to protein expression studies, some of the reviewed papers include alternative enzyme activity tests. According to these examinations, the juice or acidified 60% ethanol extracts from *Aronia melanocarpa* [115,116,119] were able to increase the activity of glucokinase (GK) and pyruvate kinase (PK) (which may enhance the glycolysis process), inhibit the activity of enzymes involved in gluconeogenesis (e.g., PEPCK, phosphoenolpyruvate carboxykinase; G6Pase, glucose-6-phosphatase), and regulate glucose homeostasis through enzymatic termination of incretin action (DPP IV inhibition). The inhibition of G6Pase was also observed for 70% methanol macerate from *Malus* sp. [122].

(5) Finally, some mechanisms that may prevent the impairment of the function of various proteins caused by high glucose levels were also revealed. For example, it was observed that the fruit extract from *Chaenomeles sinensis* [123] decreased the levels of intermediate products of glycation, i.e., glyoxal (GO) and methylglyoxal (MG). Moreover, the extract from *Crataegus orientalis* [112] was able to inhibit the activity of the ALR enzyme, which may prevent the development of diabetic complications caused by pathological glucose metabolism (polyol pathway).

The detailed information on the summarised research results can be found in Table 2.

**Table 2:** In vivo animal studies of the anti-diabetic potential of fruits from the Maleae tribe.

Species	Sample Type, Composition	Model, Study Design	Tested Parameter, Observed Effects *	Ref.
*Amelanchier alnifolia* (Nutt.) Nutt. ex M. Roem.	berry powder; anthocyanins 5011 mg/kg of dry weight (HPLC-MS: cyanidin-3-*O*-galactoside 74%, cyanidin-3-*O*-glucoside 18%, cyanidin-3-*O*-arabinoside, cyanidin-3-*O*-xyloside)	male diabetic or C57BL/J wild-type mice (n = 5–8/group); duration: 4–5 weeks; tested: diabetic or wild-type mice supplemented with berry powder (0.2, 1, 5, 20%); control: non-supplemented mice (diabetic or wild-type)	blood glucose: ↓ in diabetic mice supplemented with berry powder compared to diabetic non-supplemented mice by 17–41%, depending on the dose (the highest changes for 5% berry powder, i.e., ~8.0 g/kg/day)	[124,125,126]
	berry powder; anthocyanins 5011 mg/kg of dry weight (HPLC-MS: cyanidin-3-*O*-galactoside 74%, cyanidin-3-*O*-glucoside 18%, cyanidin-3-*O*-arabinoside, cyanidin-3-*O*-xyloside)	C57BL/6J male mice (n = 8/group); duration: 10–15 weeks; tested: mice fed a high-fat, high-sucrose diet supplemented with berry powder (1, 2.5, 5%, HFHS + B1%, HFHS + B2.5%, HFHS + B5%) or cyanidin 3-*O*-glucoside (7.2 mg/kg/day), or non-supplemented mice (HFHS); control: low-fat-diet mice	fasting plasma glucose: ↓ in supplemented mice compared to HFHS, the reduction in HFHS + B2.5% (level comparable to control) was significantly lower than in the HFHS + B1% group; plasma insulin and HOMA-IR: ↓ in supplemented mice compared to HFHS (no variation by dose); effects on gut microbiota: multiple changes, i.a., altered α-diversity and β-diversity of gut microbiota (dose ≥ 2.5%), reduced ratio of *Firmicutes/Bacteroidetes* compared to HFHS; cyanidin 3-*O*-glucoside activity: similar to *Amelanchier* berry powder containing an equal amount of cyanidin 3-*O*-glucoside	[104,105,106,107]
	berry powder; flavonoid glycosides 211.79 mg/100 g, quercetin 82.34 mg/100 g, anthocyanins 281 mg/100 g, phenolic acids 108 mg/100 g (HPLC)	male Wistar rats (n = 12/group); duration: 16 weeks; tested: corn starch or high-carbohydrate, high-fat diet + berry powder (26.83 g/kg of food = cyanidin glucoside 8 mg/kg/day) (CS + B or HFHC + B); control: corn starch (CS) or high-carbohydrate, high-fat diet (HFHC)	blood glucose: higher in HFHC and HFHC + B groups compared to CS and CS + B (dependence only on diet; no effect of fruit supplementation); OGTT: ↓ iAUC (0–120 min) of blood glucose in CS + B compared to CS, ↓iAUC in HFHC + B compared to HFHC, higher in both HFHC and HFHC + B compared to CS and CS + B (dependence on both diet and supplementation); gene expression: normalisation of HK1, GP (to a level comparable to controls), and ↑G6Pase	[121]
*Aronia melanocarpa* (Michx.) Elliott	juice; anthocyanins 5.986 nmol/mL (HPLC: cyanidin 3,5-diglucoside, cyanidin 3-*O*-arabinoside, cyanidin 3-*O*-galactoside, cyanidin 3-*O*-glucoside)	KKAy male mice (n = 5/group); duration: 49 days; tested: *Aronia* juice (A) or cyanidin 3,5-diglucoside (10 μg/mL solution); control: water-drinking mice	blood glucose: ↓ by about 61% (21 days) and by about 42% (49 days); HbA1c: ↓ by about 33% (21 days) and by about 44% (49 days); DPP IV activity in the serum: ↓ by 62% (49 days); serum active GLP-1 level: about 10-fold ↑ (49 days); cyanidin 3,5-diglucoside: similar but weaker effects compared to *Aronia* juice	[119]
	juice; proteins, carbohydrates, fats, minerals, fibres and energy density determined	C57BL/6JmsSlc or KKAy male mice (n = 5/group); duration: 28 days; tested: *Aronia*-drinking mice; control: water-drinking mice	blood glucose: ↓ by about 55% (only KKAy mice); serum insulin: ↓ (only KKAy mice); DPP IV activity in the serum and liver: ↑ (only C57BL/6JmsSlc mice); DPP IV activity in the intestine: ↓ by about 35% in the ‘upper small intestine’ and about 46% in the ‘lower small intestine’ (KKAy mice); α-glucosidase activity in the upper region of the small intestine: ↓ by about 58% (KKAy mice); expression level: ↓ GIP in the “upper small intestine” and ↑ GLP-1 expression in the “lower small intestine” (KKAy mice)	[115]
	juice; anthocyanins 9.572 mg/g, procyanidins 5.328 mg/g, flavonols 3.089 mg/g, hydroxycinnamic acids 2.71 mg/g (HPLC)	C57BL/6J male mice (n = 10/group); duration: 12 weeks; tested: low-fat, high-sucrose, and high-fat diet supplemented with *Aronia* juice concentrate (1.44 g anthocyanins/kg of diet); control: non-supplemented mice	plasma glucose and insulin: no differences between groups	[127]
	standardised *Aronia* berry extract powder (Fort Wayne, IN, USA); polyphenols 40%, anthocyanins 15%	C57BL/6J male mice (n = 10/group); duration: 14 weeks; tested: high-fat, high-sucrose diet + 0.2% (*w/w*) *Aronia* extract; control: low-fat (NC) or high-fat, high-sucrose diet (HFHS)	fasting blood glucose: ↓ compared to HFHS group; PPARγ and GLUT-4 mRNA expression levels in adipocyte fraction: ↑ compared to HFHS group (not statistically significant); anthocyanins responsible for observed effects (based on literature studies and extract composition)	[35]
	anthocyanin purified powder extract (80% EtOH + 0.1% HCl + purification); anthocyanins 986.48 mg/g (cyanidin monoglycosides)	C57BL/6J male mice (n = 10/group); STZ-induced diabetes; duration: 5 weeks; tested: high-fat diet, diabetic + 150/300 mg/kg *Aronia* extract (D/A150 and D/A300) or 200 mg/kg metformin (M); control: low-fat (NC) or high-fat diet, diabetic (DC)	blood glucose: dose-dependent ↓ compared to DC; serum insulin and HbA1c: dose-dependent ↓ compared to DC; liver glycogen: ↑ compared to DC (D/A300 and M); hepatic protein expression: ↑ *p*-GSK-3β (all groups), ↑ GLUT-4 (D/A300 and M), ↓ SOCS3, (all groups), unchanged GSK-3β and IRS-1 compared to DC; anthocyanins responsible for observed effects (testing of pure anthocyanin fraction activity)	[100]
	70% EtOH extract; detected compounds (HPLC-MS): gallic acid, chlorogenic acid, quercetin, kaempferol	male ICR mice (n = 8/group); STZ-induced diabetes; duration: 31 days; tested: *Aronia*-extract-treated diabetic mice (10 or 100 mg/kg, D/A10 or D/A100); control: normal (NC) or diabetic (DC) mice	serum glucose: ↓ compared to DC (D/A100 = 208.60 ± 31.05 mg/dL, DC mice = 486.60 ± 81.94 mg/dL, NC mice = 175.67 ± 10.60 mg/dL); serum insulin: ↑ compared to DC (D/A100 = 2.50 ± 0.39 ng/mL, DC mice = 1.34 ± 0.54 ng/mL, NC mice = 2.66 ± 0.36 ng/mL); effect on tissue injury: *Aronia* extract treatment attenuated histological changes induced by STZ in the liver and pancreatic tissues	[111]
	juice; anthocyanins 240 mg/100 mL (HPLC)	male Wistar rats (n = 12/group); duration: 16 weeks; tested: maize starch + *Aronia* juice (9.4 mg anthocyanins/kg/day) or high-carbohydrate, high-fat diet + *Aronia* juice (7.8 mg anthocyanins/kg/day); control: maize starch + water (C), high-carbohydrate, high-fat diet + water (HFHC)	OGTT: iAUC (0–120 min) of blood glucose ↑ in HFHC (786 mmol/L min), ↓ in *Aronia* groups (658 or 648 mmol/L min) compared to C rats (591 mmol/L min); plasma insulin: ↑ in HFHC (4.1 μmol/L), ↓ in *Aronia* groups (2.3 or 1.1 μmol/L) compared to C rats (1.4 μmol/L); anthocyanins responsible for observed effects (based on juice composition and literature studies)	[128]
	60% EtOH commercial extract containing at least 10% anthocyanins	male Wistar rats (n = 6/group); duration: 6 weeks; tested: fructose-rich diet + 100 or 200 mg/kg *Aronia* extract dissolved in water; control: fructose-rich diet + water	blood glucose and insulin: ↓ by about 10% (glucose) and 30% (insulin) regardless of dosage; expression of proteins: ↑ mRNA levels of IRS-1, IRS-2, PI3KR1, GLUT-1, GLUT-4, and GYS (by 1.5–2.3-fold at 200 mg/kg dosage), and ↓ mRNA levels of PTEN and GSK-3β (by 0.61–0.62-fold at 200 mg/kg)	[117]
	juice; ascorbic acid 29 g/L, anthocyanins 1.3 mg/mL, carotenes 97.8 µg/L, polyphenols 31.84 g/L	male Wistar albino rats (n = 10/group); alloxan-induced diabetes; duration: 6 weeks; tested: normal *Aronia*-drinking rats (N/A, 10 mL/kg), diabetic *Aronia*-drinking rats (D/A); control: diabetic water-drinking rats (DC), normal water-drinking rats (NC)	blood glucose: ↓ for D/A rats compared to DC rats (D/A = 136.8 ± 14.6 mg/dL, DC = 220.4 ± 23.5 mg/dL, NC mice = 103.5 ± 11.2 mg/dL)	[129]
	juice; polyphenols 709.3 mg/100 mL; flavonoids 189.4 mg/100 mL; anthocyanins 106.8 mg/100 mL, L-ascorbic acid 3.0 mg/100 ml	male Wistar rats (n = 6/group); STZ-induced diabetes; duration: 6 weeks; tested groups: normal (N/A10 and N/A20) or diabetic (D/A10 and D/A20) rats drinking *Aronia* juice (10 or 20 mL/kg); control: normal (NC) or diabetic (DC) rats drinking water (10 mL/kg)	plasma glucose: DC rats = 17.5 ± 2.9 mmol/l; NC rats = 7.2 ± 0.6 mmol/l; N/A10 and N/A20 rats—no significant changes compared to NC rats; D/A10 and D/A20 rats—↓ to levels comparable to NC rats (by 44% and 42% compared to DC rats, respectively); anthocyanins and flavonoids responsible for observed effects (based on juice composition and literature studies)	[130]
	commercial extract; polyphenols 714.1 mg/g, including anthocyanins 56.6% (cyanidin monoglycosides), flavanols 21.6% (procyanidins, epicatechin), phenolic acids 14.7% (chlorogenic and neochlorogenic acids), flavonols 7.1% (quercetin glycosides) (HPLC)	male Wistar rats (n = 8/group); STZ-induced diabetes; duration: 4 weeks; tested: diabetic rats with diet modified by 8% lard and 65% fructose + *Aronia* extract (0.2%); control: diabetic (DC) or normal rats (NC) fed a standard casein diet enriched with 0.5% cholesterol	activity of microbial enzymes in the caecal digesta: β-glucuronidase activity ↑ in the DC group compared to NC, and ↓ in the *Aronia* group to a level no different vs. NC; the activity of α- and β-glucosidase and α- and β-galactosidase did not differ; mucosal disaccharidase activities: sucrase and maltase activity comparable between the NC and *Aronia* groups and ↑ in DC; in the case of lactase the trend was the opposite; serum glucose: no differences between the *Aronia* and NC groups, remarkably ↑ in the DC group	[114]
	60% EtOH: 0.1% HCl (1:20) extract, purified and concentrated; anthocyanins 254.72 mg/g (cyanidin monoglycosides), flavonoids 11.66 mg/g (quercetin glycosides), phenolics acids 75.97 mg/g (caffeic, chlorogenic, and neochlorogenic acids) (HPLC-MS)	male Wistar rats (n = 6/group); STZ-induced diabetes; duration: 8 weeks; tested: diabetic rats fed a high-fat diet and 100 or 400 mg/kg *Aronia* extract (D/A100 or D/A400); control: normal rats, standard diet (NC) and diabetic rats, high-fat diet (DC)	OGTT: iAUC (0–120 min) of blood glucose ↓ by 24.40% in the D/A400 rats compared to DC rats; blood glucose: ↓ by 2.76 and 4.25 mmol/L compared to DC rats; serum insulin: ↓ compared to DC rats (statistically insignificant); HOMA-IR: 3.26 ± 0.56 (NC), 15.42 ± 4.17 (DC), 11.24 ± 3.10 (D/A100), 8.12 ± 1.94 (D/A400); hepatic glycogen: ↑ compared to DC rats; liver enzyme activity: GK and PK ↑ compared to DC rats, PEPCK and G6Pase ↓ compared to DC rats; hepatic protein expression: ↑ *p*-IRS-2, *p*-PI3K, *p*-Akt, *p*-GSK-3β, and GLUT-2 (by 2.03–4.02-fold for D/A400), and ↓ IRS-2 and GSK-3β (by 1.53–2.76-fold for D/A400), compared to DC	[116]
	water extract; anthocyanins 579.1 mmol/g (cyanidin monoglycosides), (+)-catechin 10.7 mmol/g, chlorogenic acid 32.7 mmol/g, caffeic acid 13.9 mmol/g (HPLC)	male Sprague-Dawley rats (n = 6/group); duration: 4 weeks; tested: high-fat diet + *Aronia* extract (17.4 g extract/kg of diet); control: non-supplemented rats	serum glucose: ↓ (8.20 ± 0.31 mM/dL) compared to controls (9.17 ± 0.38 mM/dL); OGTT: iAUC (0–120 min) of blood glucose ↓ by 20.27%; anthocyanins, proanthocyanidins, and chlorogenic acid responsible for observed effects (based on extract composition and literature studies)	[131]
	pomace	Polish Merino health lambs (n = 8/group); duration: 90 days; tested: 150 g or 300 g (A150 or A300) of *Aronia* pomace/kg of the feed mixture (twice a day, intake monitored and adjusted to the lambs’ growing period); control: non-supplemented lambs	serum glucose: A150 = 2.42 ± 0.31 mmol/L, A300 = 1.55 ± 0.66 mmol/L, control = 3.38 ± 0.31 mmol/L; total phenolic contents in the liver or serum: A150 = 7.49 mg GAE/g of liver and 2.41 mg GAE/mL of serum; A300 = 7.46 mg GAE/g of liver and 3.93 mg GAE/mL of serum; control = 4.42 mg GAE/g of liver and 1.77 mg GAE/mL of serum	[132]
*Chaenomeles sinensis* (Thouin) Koehne	EtOH extract with removed sugars; polyphenols 350 mg GAE/g, including procyanidins (>90%)	KKAy male mice (n = 4–5/group); duration: 4 weeks; tested: diabetic mice fed a high-fat diet + extract (14.3 g/kg diet); control: diabetic mice fed a high-fat diet (DC)	OGTT: ↓ blood glucose after 15 min, iAUC (0–120 min) unchanged; blood glucose: ↓ after 1–4 weeks; HbA1c: unchanged (↓, but not significantly); intermediate products of glycation: 3-DG level not affected, GO and MG levels ↓ about twofold; procyanidins responsible for observed effects (based on extract composition and literature studies)	[123]
*Crataegus azarolus var. aronia* L.	water decoction of unripe fruits	female Sprague-Dawley rats (n = 10/group); STZ-induced diabetes; duration: 24 days; tested: diabetic (D) or normal (N) rats + *Crataegus* decoction of 50 or 350 mg fruits/mL (D50, D350, N50, N350); controls: normal rats (NC) and diabetic rats (DC)	blood glucose: ↓ in N350 compared to NC rats, ↓ in D50 and D350 compared to DC rats	[133]
*Crataegus laevigata* (Poir.) DC.	70% EtOH extract	Sprague-Dawley rats (n = 12/group); STZ-induced diabetes; duration: 6 h; tested: diabetic rats + 200, 400, 600, 800, 1000, 1200 mg/kg *Crataegus* extract; controls: normal rats (NC), diabetic rats (DC), and diabetic rats + glipizide (10 mg/kg)	blood glucose: dose-dependent ↓ compared to DC, hypoglycaemic effect of *Crataegus* extract at 1200 mg/mL comparable to glipizide; OGTT: effective ↓ in glucose levels after 30, 60, and 90 min (dosage 800 and 1200 mg/mL)	[101]
*Crataegus meyeri* Pojark.	water extract from fresh pulp	male Wistar rats (n = 10/group); STZ-induced diabetes; duration: 10 weeks; tested: diabetic rats + 300 mg/kg *Crataegus* extract; controls: normal rats (NC) and diabetic rats (DC)	insulin level and QUICKI: unchanged; glucose: ↓ compared to DC (NC 118 ± 32 mg/dL, DC 538 ± 47 mg/dL, *Crataegus* 348 ± 41 mg/dL); HOMA-IR: ↓ (NC 130 ± 19, DC 177 ± 16, *Crataegus* 119 ± 21)	[134]
*Crataegus monogyna* Jacq.	70% MeOH extract	male Wistar rats (n = 8/group); STZ-induced diabetes; duration: 3 weeks; tested: diabetic rats + 100, 200 or 400 mg/kg *Crataegus* extract; controls: normal rats (NC) and diabetic rats (DC)	serum glucose: ↓ compared to DC rats (to levels comparable to NC; all dosages); effects on pancreatic tissue: *Crataegus* extract (at 200 and 400 mg/kg) ameliorated the degeneration of pancreatic acinar cells; damage to lobules, acini, and oedema observed in the tissue of DC rats	[135]
*Crataegus orientalis* subsp. *presliana* K.I.Chr.	90% EtOH extract	Kunming mice (n = 8/group); STZ-induced diabetes; duration: 4 weeks; tested: diabetic mice + extract 1.8 g/kg; controls: normal (NC) or diabetic mice (DC)	blood glucose: ↓ compared to DC (statistically insignificant); ALR activity: ↓ compared to DC (statistically insignificant)	[112]
*Crataegus pinnatifida* Bunge	proanthocyanidin fraction from 70% EtOH extract; procyanidins 81.85 mg/100 mg (LC-MS: epicatechin 36.12%; procyanidins B2, B5, and C1)	male Wistar rats (n = 12/group); duration: 8 weeks; tested: high-fat diet + 50, 100, or 200 mg/kg *Crataegus* preparation; controls: standard diet (NC) or high-fat diet (HFD)	fasting blood glucose: lack of differences (tested and control groups); OGTT: glucose level ↓, iAUC (0–120 min) ↓ compared to HFD; insulin: dose-dependent ↓ compared to HFD; alleviation of liver histopathological changes compared to HFD; procyanidins responsible for observed effects (based on activity testing of extract fraction)	[110]
	MeOH extract; total phenolics 66.94 mg GAE/g	male Sprague-Dawley rats (n = 9/group); STZ-induced diabetes; duration: 2 weeks; tested: diabetic rats fed a high-fat diet + *Crataegus* extract at 50, 100, or 200 mg/kg; controls: normal rats, standard diet (NC); diabetic rats, high-fat diet (DC); diabetic rats, high-fat diet + orlistat (40 mg/kg)	blood glucose: dose-dependent ↓ compared to DC; 5.62 ± 0.39 mmol/L (NC), 20.25 ± 1.9 mmol/L (DC), 10.5—17.9 mmol/L (*Crataegus*), about 10 mmol/L (orlistat); serum insulin: dose-dependent ↓ compared to DC; 147.74 ± 15.61 pg/mL (NC), 238.59 ± 21.01 pg/mL (DC), 176.82—206.76 (*Crataegus*), about 300 pg/mL (orlistat)	[136]
*Crataegus pinnatifida var. major* N.E.Br.	70% acidic EtOH (0.1% HCl) extract; polyphenols 594.15 mg/g, including chlorogenic acid 15.2%, procyanidin B2 20.3%, epicatechin, proanthocyanidin B-type oligomers, cyanidin 3-galactoside, and quercetin glycosides (HPLC-MS)	male Wistar rats (n = 8/group); STZ-induced diabetes; duration: 12 weeks; tested: diabetic rats fed a high-fat diet + 300 mg/kg *Crataegus* extract; controls: normal rats, standard diet (NC); diabetic rats, high-fat diet (DC); diabetic rats, high-fat diet + metformin (150 mg/kg)	blood glucose: 44.2% ↓ compared to DC rats (likewise for metformin); OGTT: glucose level ↓ after 30 min (17.32 mmol/L) compared to DC rats (25.80 mmol/L), comparable to metformin (15.73 mmol/L); serum insulin: 34.1% ↓ compared to DC rats (about 50% reduction for metformin); improved histology of skeletal muscle, liver, and aortic vessels; phosphorylation of proteins: ↑ *p*-GLUT-4, *p*-IRS-1, *p*-Akt, and *p*-PI3K in the liver; ↑ *p*-IRS-1 and *p*-Akt but no effects on *p*-PI3K in the skeletal muscle (comparable to metformin)	[102]
	*n*-butanol fraction of 80% MeOH extract	C57BL/6J male mice (n = 5–9/group); duration: 8 weeks; tested: high-fat diet + *Crataegus* extract at 200, 500, or 1000 mg/kg (CE200–1000); controls: low-fat diet (NC), high-fat diet (HF), and high-fat diet + rosiglitazone (10 mg/kg, Rosi10)	plasma glucose and insulin: ↓ compered to HF, comparable to NC in the CE1000 and Rosi10 groups; insulin resistance test: ↓ compared to HF (significantly in the CE500, CE1000, and Rosi10 groups); GLUT-4 expression (skeletal muscle): the mRNA level ↓ in HF and ↑ in CE200–1000 and Rosi10 compared to NC; protein expression in the liver: ↓ PEPCK (CE500, -1000, and Rosi10) compared to HF, ↑ *p*-AMPK (all groups) compared to NC and HF; OGTT: ↓ in glucose levels after 30 min in low-fat diet mice supplemented with 200 mg/kg extract compared to NC, and after 30, 60, 90, 120, and 180 min in mice supplemented with 1000 and 2000 mg/kg	[118]
*Crataegus* L. sp.	water extract from dried pulp; polyphenols 20.18 mg GAE/g, flavonoids 8.50 mg QE/g	male Wistar rats (n = 6–8/group); STZ-induced diabetes; duration: 14 days; tested: diabetic rats + extract at 100, 300, or 1000 mg/kg; controls: normal rats (NC) and diabetic rats (DC)	blood glucose: ↓ compared to DC at 100 and 300 mg/kg; unchanged at 1000 mg/kg	[137]
	water extract from dried pulp	male Wistar rats (n = 10/group); STZ-induced diabetes; duration: 10 weeks; tested: diabetic rats + extract at 100 mg/kg ± resistance training; controls: normal rats (NC) and diabetic rats (DC)	blood glucose: unchanged compared to DC; fastening serum insulin: ↑ compared to DC (NC 25.0 μIU/mL; DC 6.93 μIU/mL, extract 8.12 μIU/mL)	[138]
	80% EtOH extract; detected compounds (HPLC): chlorogenic acid, catechin, epigallocatechin gallate, quercetin, kaempferol and apigenin derivatives (including hyperoside and vitexin)	male Wistar rats (n = 8/group); STZ-induced diabetes; duration: 12 weeks; tested: diabetic rats + extract at 100 mg/kg ± resistance training; controls: normal rats (NC) and diabetic rats (DC)	blood glucose: ↓ compared to DC (NC 6.78 mmol/L; DC 22.98 mmol/L, extract 17.96 mmol/L); serum insulin: ↑ compared to DC (NC 10.25 μU/mL; DC 4.98 μU/mL, extract 7.67 μU/mL); GPLD1 serum level: ↓ compared to DC; GPC-4 serum level: ↑ compared to DC (statistically insignificant); HOMA-IS: ↑ compared to DC (statistically insignificant)	[120]
*Cydonia oblonga* Mill.	30% EtOH extract; chlorogenic acid 0.75 mg/g (HPLC)	C57BL/6N mice (n = 10/group); duration: 8 weeks; tested: high-fat diet + extract at 50, 100, or 200 mg/kg; controls: normal diet (NC) and high-fat diet (HFD),	serum glucose: unchanged; serum insulin: ↓ compared to HFD (NC 2.62 ng/mL, HFD 9.12 ng/mL, *Cydonia* 5.67–6.51 ng/mL); HOMA-IR: ↓ compared to HFD (all dosages); QUICKI: ↑ compared to HFD at 200 mg/kg; *p*-AMPK/AMPK ratio ↑ about twofold compared to HFD (200 mg/kg), PPARγ mRNA expression ↓ about twofold (all dosages) chlorogenic acid responsible for observed effects (based on extract composition and literature studies)	[108]
*Malus domestica* (Suckow.) Borkh.	commercial extract (Biosearch S.A.); polyphenols 80%, phlorizin min. 5% (HPLC)	male Wistar rats (n = 12/group); duration: 56 days; tested: rats fed a high-fat, high-sucrose diet + extract at 700 mg/kg (HFS + M); controls: standard diet (NC) and high-fat, high-sucrose diet (HFS)	serum glucose: ↓ compared to HFS (HFS + M 5.50 ± 0.22 mmol/L, HFS 6.36 ± 0.28 mmol/L, NC 5.45 ± 0.23 mmol/L); serum insulin: ↓ compared to HFS (HFS + M 14.6 µU/mL, HFS 22.9 µU/mL, NC 9.12 µU/mL); HOMA-IR: ↓ compared to HFS (HFS + M 3.45, HFS 6.65, NC 2.18)	[109]
	juice and peel water extracts; polyphenols 69.68 ± 2.17 mg GAE/100 mL (juice), 673.46 ± 6.90 mg GAE/ 100 g dry extract	male Wistar rats (n = 8/group); STZ-induced diabetes; duration: 21 days; tested: diabetic rats + apple juice (15 mL/kg) or peel extract (1 g/kg); controls: normal rats (NC) and diabetic rats (DC)	blood glucose: ↓ compared to DC after the first, second, and third weeks, to levels comparable to NC (juice: 87.87–134.83 mg/dL, extract: 88.00–105.82 mg/dL, DC: 374.67–432.14 mg/dL, NC: 81.53–89.57 mg/dL)	[139]
	extract dissolved in water	Wistar rats (n = 5/group); fructose-induced diabetes; duration: 28 days; tested: diabetic rats + apple extract (500 mg/kg); controls: normal rats (NC) and diabetic rats (DC)	blood glucose: ↓ compared to DC, to levels comparable to NC (apple: 81.00 ± 3.61 mg/dL, DC: 116.70 ± 9.13 mg/dL, NC: 76.33 ± 3.84 mg/dL)	[140]
	commercial extract (BioActive Food GmbH); polyphenols 44% (catechin equivalents), phlorizin 16%, quercetin 12.43%, chlorogenic acid 5.57% (HPLC/spectrophotometry)	male C57BL/6N mice (n = 10/group); duration: acute consumption; tested: mice fed a high-fat diet + 12.24 mg of extract or 1.96 mg of phlorizin; controls: normal diet (NC) and high-fat diet (HFC)	OGTT: iAUC (0–15, 0–30, 0–60 min) ↓ compared to HFC (to levels comparable to NC), with the highest reduction after 15 min (likewise for phlorizin); glucose uptake in everted jejunal rings: inhibited (EC_50_ = 8.9 ± 2.2 µg/mL for extract and 4.2 ± 0.6 µM for phlorizin); glucose uptake (everted jejunal sacs): SGLT1 inhibited (reversible manner)	[75]
	peel extract, purified, sugar-free; polyphenols 606 mg GAE/g (HPLC: chlorogenic acid, epicatechin, quercetin glycosides), anthocyanins 7.17 mg/g	C57BL/6J male mice (n = 8/group); duration: 10 weeks; tested: high-fat diet + 0.2% (*w/w*) extract or quercetin; controls: low-fat diet (NC) and high-fat diet (HFC)	fasting blood glucose: ↓ compared to HFC (comparable to NC); IPGTT: ↓ iAUC (0–2 h) of blood glucose compared to HFC; serum insulin: ↓ compered to HFC; activity of quercetin: comparable to apple extract	[141]
	juice (fermented or not); compounds: 19 polyphenols (HPLC: mainly chlorogenic acid about 95–145 mg/L, epicatechin 70–105 mg/L, procyanidin B2 70–95 mg/L), sugars, and organic acids	C57BL/6J mice; STZ-induced diabetes; duration: 4 weeks; tested: high-fat diet + juice (fermented or unfermented); controls: normal mice, normal diet (NC); diabetic mice, high-fat diet (DC); diabetic mice, acarbose (A)	fasting blood glucose: ↓ compared to DC (both fermented and unfermented juice), for fermented juice and acarbose to levels comparable to NC; insulin and HOMA-IR: similar trend as for glucose levels; QUICKI: ↑ compared to DC (fermented and unfermented juice), for fermented juice and acarbose to levels comparable to NC	[82]
*Malus* Mill. sp.	70% MeOH macerate	albino mice; duration: 22 days; tested: plant extract 1 g/kg, 2 g/kg, or 3 g/kg; control: non-supplemented mice	G6Pase activity: dose-dependent inhibition (activity at 1 g/kg = 20.7–22.0, at 2 g/kg = 10.4–13.4, at 3 g/kg = 1.3–2.4, compared to control = 70.35), higher than observed for, e.g., mulberry fruit extract	[122]
*Malus pumila* Mill.	procyanidin fraction from juice (without chlorogenic acid and phlorizin)	male B6.Cg-Lepob/J mice (C57BL/6J background) (n = 6/group); obese, insulin-resistant, moderate hyperglycaemia; duration: 4 weeks; tested: 0.5% extract dissolved in water, ad libitum, control: non-supplemented obese mice	OGTT (0–120 min): blood glucose ↓ at 15 and 30 min, serum insulin unchanged; insulin TT (0–120 min): blood glucose ↓ at 15, 30, 45, and 60 min; HOMA-IR: ↓ (27.3 ± 7.9) compared to control mice (76.0 ± 13.3); pancreatic islet size: β-cell area ↓ by about 21%, number of islets unchanged (↓ in pancreatic cells’ hypertrophy); pyruvate TT: glucose ↓ at 15–30 min (↓ in hepatic gluconeogenesis); protein phosphorylation: ↑ *p*-Akt; suppression of hepatic inflammation resulting in insulin signalling improvement	[113]
	commercial extract (Exxentia^®^); polyphenols 57.5%, including phlorizin (9.9%), chlorogenic acid (15.8%), quercetin (0.4%)	obese male Zucker fatty rats—insulin-resistant model (n = 10/group); duration: acute consumption (for TT) and 4 weeks; tested: (TT) for first meal maltodextrin + 150 mg extract/kg, for second meal maltodextrin only, (chronic) standard diet + 128 mg extract/kg; control: (TT) maltodextrin, (chronic) standard diet	acute meal TT: iAUC (0–120 min) ↓ for glucose and unchanged for insulin; second acute meal TT: iAUC (0–240 min) for glucose ↓, glucose levels higher compared to tested rats after first meal; chronic effect on insulin sensitivity: iAUC (0–180) ↓ for both glucose and insulin levels, insulin sensitivity ↑ (glucose infusion rate required to establish euglycaemia ↑ by 45%)	[30]
*Pyrus* *bretschneideri* Rehder	60% MeOH extract from peel and pulp; polyphenols about 2.9/8.1 mg GAE/g pulp/peel (HPLC-MS: catechin; epicatechin; rutin; chlorogenic, *p*-coumaric, vanillic, gallic, and ferulic acids), flavonoids about 1.5/6.3 mg RE/g, terpenes (oleanolic and ursolic acids) about 0.9/4.3 mg OAE/g	male Kunming mice (n = 10/group); STZ-induced diabetes; duration: 3 weeks; tested: diabetic mice, high-fat diet + 500 mg/kg peel or pulp extract; controls: normal mice, normal diet (NC); diabetic mice, high-fat diet (DC)	blood glucose: ↓ (8.2–8.6 mmol/L) in the peel group after 2–3 weeks compared to DC (14.7–16.0 mmol/L); OGTT: ↓ blood glucose in the peel group compared to DC, ↓ iAUC (0–3 h)	[90]
*Pyrus communis* L.	EtOAc and 80% EtOH extracts; phytochemical screening: carbohydrates, phenolics, tannins, and flavonoids	Wistar rats (n = 6/group); dexamethasone-induced diabetes; duration: 11 days; tested: diabetic rats + 200 mg/kg EtOAc or 80% EtOH; controls: normal rats (NC), diabetic rats (DC), and diabetic rats + glibenclamide (5 mg/kg)	OGTT: ↓ blood glucose after 60 min compared to DC (comparable to glibenclamide); urine sugar: significant levels in DC rats, trace amounts in tested groups and glibenclamide controls; blood glucose: ↓ from 3rd to 11th day compared to DC (comparable to glibenclamide)	[103]
*Pyrus pyrifolia* (Burm.f.) Nakai	80% EtOH + 70% acetone extract from different cultivars; polyphenols 20.9–28.5 mg CE/g	male DDY mice (n = 8/group); duration: acute consumption; tested: 250 or 500 mg/kg Pyrus extract; control: non-supplemented mice	oral starch TT: ↓ blood glucose after 30 min at 250 mg/kg and after 30, 60, and 120 min at 500 mg/g; ↓ iAUC (2 h) at 500 mg/kg	[96]
	50% EtOH extract from pomace	C57BL/6J male mice (n = 10/group); duration: 8 weeks; tested: high-fat diet + 200 or 400 mg/kg extract; control: high-fat diet + water	blood glucose: unchanged; insulin: ↓ at 400 mg/kg (not significantly) HOMA-IR: ↓ at 400 mg/kg; protein expression: ↑ *p*-IRS-1 (Tyr632), ↑ GLUT-4, ↓ *p*-IRS-1 (Ser307)	[31]
*Sorbus* *aucuparia* L.	EtOH extract	Kunming mice (n = 8/group); STZ-induced diabetes; duration: 4 weeks; tested: high-glucose, high-fat diet mice + extract at 10, 50, or 100 mg/L; controls: normal-diet mice (NC) and high-glucose, high-fat-diet mice (HGFD)	blood glucose: ↓ in the extract-fed mice compared to HGFD (dose-dependent, about twofold ↓ at 100 mg/L); IPGTT: ↓ serum glucose in the extract-fed mice compared to HGFD (dose-dependent, at 50 mg/L significant ↓ after 15 min, at 100 mg/L significant ↓ after 5–120 min)	[142]
*Sorbus norvegica* Hedl.	80% acetone extract; detected compounds (NMR): chlorogenic and neochlorogenic acids, carbohydrates	C57BL/6J male mice (n = 6–10/group); STZ-induced diabetes; duration: acute consumption (3.5 h); tested: mice fed starch (2 g/kg) and berry extract (600, 900, or 1250 mg/kg) or mice fed glucose (2 g/kg) and berry extract (1250 mg/kg); controls: mice fed starch and acarbose (25 mg/kg, positive control) or mice fed starch/glucose (negative control)	oral starch TT: ↓ maximal blood glucose compared to negative controls; the activity of berry extract at 900–1250 mg/kg was comparable to that of acarbose; for extract at 1250 mg/kg, ↓ iAUC; OGTT: for extract at 1250 mg/kg, ↓ in blood glucose after 30 min compared to the negative control	[22]

* Suggestions of what compounds were responsible for the observed effects were taken directly from the cited papers; for a critical discussion, see Section 3.4. ↑, increase; ↓, decrease; 3-DG, 3-deoxyglucosone; Akt, protein kinase B; ALR, aldose reductase; C3G, cyaniding 3-O-glucoside; CE, catechin equivalents; DPP IV, dipeptidyl peptidase IV; EtOAc, ethyl acetate; EtOH, ethanol; G6Pase, glucose-6-phosphatase; GAE, gallic acid equivalents; GIP, glucose-dependent insulinotropic polypeptide; GK, glucokinase; GLP-1, glucagon-like peptide 1; GLUT-1, glucose transporter 1; GLUT-2, glucose transporter 2; GLUT-4, glucose transporter-4; GO, glyoxal; GP, glycogen phosphorylase; GPC-4, glypican-4; GPLD1, insulin-regulated glycosylphosphatidylinositol-specific phospholipase D; GSK-3β, glycogen synthase kinase 3 beta; GYS, glycogen synthase; GYS2, glycogen synthase 2; HbA1c, glycated haemoglobin; HK1, hexokinase 1; HOMA-IR, the homeostasis model assessment-estimated insulin resistance; HOMA-IS, the homeostasis model assessment of insulin secretion; HPLC, high-performance liquid chromatography; HPLC-MS, high-performance liquid chromatography coupled with mass spectrometry; iAUC, incremental area under the curve; ICR, Institute of Cancer Research; IPGTT, intraperitoneal glucose tolerance test; IRS-1, insulin receptor substrate 1; IRS-2, insulin receptor substrate 2; LC-MS, liquid chromatography coupled with mass spectrometry; MeOH, methanol; MG, methylglyoxal; OAE, oleanolic acid equivalents; OGTT, oral glucose tolerance test; *p*-Akt, phosphorylated Akt; *p*-AMPK, phosphorylated AMP-activated protein kinase; *p*-GLUT-4, phosphorylated GLUT-4; PEPCK, phosphoenolpyruvate carboxykinase; PFK1, phosphofructokinase 1; *p*-GSK-3β, phosphorylated GSK-3β; PI3K, phosphoinositide 3-kinase; PI3KR1, phosphatidylinositol 3 kinase regulatory subunit 1; *p*-IRS-1, phosphorylated IRS-1; *p*-IRS-2, phosphorylated IRS-2; PK, pyruvate kinase; PPARγ, peroxisome proliferator-activated receptor gamma; *p*-PI3K, phosphorylated PI3K; PTEN, phosphatase and tensin homolog; QUICKI, quantitative insulin sensitivity check index; RE, rutin equivalents; SGLT1, sodium–glucose transporter; SOCS3, suppressor of cytokine signalling 3; STZ, streptozotocin; TT, tolerance test.

### 3.3. Human Studies

The in vivo human studies on the anti-diabetic potential of fruits from the Maleae tribe referred to five species, i.e., *Aronia melanocarpa, Malus domestica*, *Malus sylvestris*, *Malus pumila,* and *Pyrus pyrifolia* (Table 3).

The 1–3-month *Aronia melanocarpa* juice or extract supplementation resulted in reduced fasting blood glucose and glycated haemoglobin (HbA1c) levels in diabetic patients [143,144,145]. Moreover, the study of Simeonov et al. [143] showed that 60 min after ingestion of *Aronia* juice, the blood glucose levels in diabetic patients were reduced; however, this effect was statistically insignificant when the *Aronia* supplementation was combined with the meal. On the other hand, it was suggested that one-time *Aronia* juice supplementation decreased the postprandial blood glucose excursion in healthy people [146].

The anti-diabetic potential of *Malus domestica* fruits was tested only on healthy adults during one-time consumption of the apple powder or commercial apple extracts with the carbohydrate meal (postprandial response tests) or glucose (oral glucose tolerance test (OGTT)) [32,34,73,75,147]. These trials indicated the delay time (T_max_) and the lower or unchanged maximal glucose and insulin levels (C_max_), as well as the reduction in the total glucose concentration during the time of the study (iAUC, incremental area under the curve of glycaemic excursion). Moreover, a significant decrease in the glucose-dependent insulinotropic peptide (GIP) and C-peptide (an indicator of pancreatic β-cell function) was observed. The urinary glucose excretion was increased or unchanged.

The positive effects on the OGTT parameters (decreased iAUC and glucose levels after 30 min) [148,149] or postprandial response to carbohydrate meals (decreased iAUC and C_max_) [148,149] were also observed for *Malus pumila* and *Pyrus pyrifolia*. In the first study, the effect was significant in the patients with high–normal or borderline glucose levels (100–125 mg/dL) who were regularly supplemented with the apple extract for 12 weeks. In the second study, the participants with normal glucose levels received a single apple or pear preload combined with specialised carbohydrate meals. Finally, the fasting blood glucose levels were significantly reduced in diabetes mellitus type II patients who consumed *Malus sylvestris* fruits for 14 days [150].

**Table 3:** Human studies of the anti-diabetic potential of fruits from the Maleae tribe.

Species	Sample Type, Composition	Model, Study Design	Tested Parameter, Observed Effects *	Ref.
*Aronia melanocarpa* (Michx.) Elliott	juice; proteins, carbohydrates, fats, minerals, fibres, and energy density determined	open-label, randomised, two-period, one-way crossover study; 37 healthy Japanese patients (women and men, >30 years old); 100 mL of *Aronia* juice (tested) or 100 mL of water (control) + 200 g of rice; duration: acute consumption	postprandial blood glucose: ↓ iAUC (0–150 min) by > 50%	[146]
	juice; low-calorie, sugar-free	(1) 41 diabetic patients (16 insulin-dependent, 25 non-insulin-dependent, women and men, 3–62 years old); glucose at baseline (control) vs. 60 min after ingestion of 200 mL of juice or 200 mL of juice and standard meal (tested); duration: acute consumption; (2) 21 diabetic patients (non-insulin-dependent, women and men, 42–62 years old) drinking 200 mL of juice daily (tested) and 23 diabetic patients (non-insulin-dependent, women and men, 48–67 years) without supplementation (controls); duration: 3 months	fasting blood glucose (1): ↓ from 14.23 ± 1.32 mmol/L at baseline to 11.4 ± 0.89 mmol/L after 60 min; postprandial blood glucose (1): ↓ (statistically insignificant); blood glucose (2): ↓ from 13.28 ± 4.55 mmol/L at baseline to 9.10 ±3.05 mmol/L after 3 months; HbA1c (2): ↓ from 9.39 ± 2.16% at baseline to 7.49 ± 1.33% after 3 months	[143]
	juice; total phenolics 413.0 ± 5.1 mg GAE/100 g of fresh fruits, total anthocyanins 172.7 ± 4.4 mg/100 g	35 diabetic patients (women and men, 35–65 years old, diabetes type 2); 3 × 50 mL per day of *Aronia* juice (tested), and the same patients not supplemented with *Aronia* juice (control); duration: 3 months for supplementation, next 3 months for self-control	fasting blood glucose: ↓ (statistically insignificant); HbA1c: ↓ from 59.1–59.4 mmol/mol (baseline and control) to 55.1 ± 14.7 mmol/mol	[144]
	juice	11 overweight and 11 normal-weight patients (women and men, 51.9 ± 3.9 years old); 3 × 50 mL per day of *Aronia* juice (tested) and baseline parameters (control); duration: 3 months	fasting blood glucose and HbA1c: not changed	[151]
	standardised commercial extract (Alixir 400 PROTECT^®^, polyphenols (431 mg/30 mL), anthocyanins (120 mg/30 mL), potassium sorbate (35.1 mg/30 mL)); detected compounds (HPLC): cyanidin and quercetin glycosides	prospective, open-label, clinical case-series study; 143 patients with metabolic syndrome, with or without diabetes type 2 (women and men, 50–60 years old); 30 mL of *Aronia* extract per day, control: baseline parameters; duration: 28 days	blood glucose: ↓, especially in diabetic groups (6.40–6.82 mmol/L) compared to baseline levels (7.97–8.41 mmol/L)	[145]
*Malus domestica* (Suckow) Borkh.	standardised commercial extract (Appl’In by DIANA Food SAS); total polyphenols 67%, including 40% flavonoid monomers and phenolic acids (HPLC: flavan-3-ols, dihydrochalcones, flavonols, hydroxycinnamic acids)	randomised, controlled, double-blind crossover study; 20 healthy men and 5 postmenopausal women (20–60 years old); 200 mL drink containing 1200 mg of apple phenolics (tested) or <5 mg of phenolics (control) + a high-carbohydrate meal; duration: acute consumption	postprandial plasma glucose: ↓ iAUC (0–30 min), ↓ C_max_, ↑ T_max_; postprandial insulin: ↓ iAUC (0–30 min and 0–120 min), ↓ at 10, 20, 30, and 45 min, ↑ T_max_; C-peptide: ↓ iAUC (0–30 min), ↓ at 10, 20, 30, and 45 min, ↑ T_max_; GIP: ↓ iAUC (0–30 min and 0–120 min), ↓at 10, 20, 30, 45, 60, and 75 min, ↓ C_max_ decreased, ↑ T_max_;	[32]
	standardised commercial extract (Appl’In, Diana Naturals, France); min. 80% polyphenols, >5% phlorizin	randomised controlled trial, balanced incomplete block design; 65 healthy adults (women and men, average 20–50 years old), apple extract group: 33 adults; 2 g of plant extract (tested) or not supplemented (control) + rice porridge (~50 g available carbohydrate); duration: acute consumption	postprandial glucose: ↓ iAUC (0–2 h) by about 25.4%, effect comparable to those of mulberry fruit and leaf extracts; postprandial insulin: ↓ iAUC (0–2 h) by about 22.3% urine glucose: no glucosuria observed	[34]
	standardised commercial extract (Appl’In, Diana Naturals, France); polyphenols min. 80%, phlorizin > 5%; 14 compounds detected (HPLC), including quercetin glycosides, dihydrochalcones, phenolic acids, and procyanidin oligomers	randomised, controlled, double-blind crossover study; 30 healthy adults (women and men, 18–68 years old); drink without (control) or with 1.8, 1.35, or 0.9 g of apple extract (tested) + a 75 g starch/sucrose meal; duration: acute consumption	postprandial blood glucose: ↓ iAUC (0–30 min) by about 8.99–15.6 mmol/L per minute (all doses), iAUC (0–120 min and 0–240 min) unchanged, ↑T_max_, C_max_ unchanged; postprandial insulin and C-peptide: similar trends as for glucose concentration; GIP: ↓ at 30 and 60 min (1.8 and 1.35 g), T_max_ and C_max_ unchanged; urinary glucose: unchanged different compounds responsible for observed effects (based on literature studies, extract composition, and serum metabolite studies)	[73]
	apple powder from unripe apples; total sugars 153.44 g/kg, water-soluble pectins 27.73 g/kg, phlorizin 12.61 g/kg, chlorogenic acid 18.90 g/kg, catechin, epicatechin, quercetin glycosides, phloretin-2-*O*-D-xyloglucoside (HPLC)	open-label, randomised crossover study; six healthy females with increased risk of cardiovascular disease and diabetes; 25 g of apple preparation (tested) or not supplemented (control) + glucose; duration: acute consumption	OGTT: glucose at 15 to 30 min reduced ↓ by about twofold, urinary glucose excretion after 2–4 h ↑ by about fivefold; phlorizin responsible for observed effects (based on extract composition and urine metabolite studies)	[147]
	commercial extract (BioActive Food GmbH); total polyphenols 44% (catechin equivalents), phlorizin 16%, quercetin 12.43%, chlorogenic acid 5.57% (HPLC/spectrophotometry)	randomised crossover study; 10 healthy men (23.5 ± 3.1 years old); 2.8 g of capsuled apple extract (tested) or not supplemented (control) + glucose; duration: acute consumption	OGTT: ↓ iAUC (0–15, 0–30, and 0–45 min), glucose level at 15, 30, and 45 min timepoints not significantly changed insulin: ↓ iAUC (0–30, 0–45, 0–60, and 0–90 min), with the highest reduction after 30 min; urinary glucose: 4.9-fold higher in the first 3 h	[75]
*Malus pumila* Mill.	polyphenolic extract; 48.9% procyanidins (dimers, trimers, tetramers, pentamers, hexamers, and polymers), 14.1% flavan-3-ols (monomers) and 10.5% phloretin glucosides, including phlorizin (HPLC)	double-blinded, placebo-controlled study; 65 patients (30–60 years old) with normal (<100 mg/dL), high–normal (100–109 mg/dL), and borderline (110–125 mg/dL) glucose; 600 mg of apple polyphenols/day (tested) or placebo (control); duration: 12 weeks	OGTT: glucose ↓ after 30 min in the high–normal and borderline groups (164.0 ± 7.4 mg/dL) compared to controls (194.7 ± 10.4 mg/dL), ↓ iAUC (0–2 h), normal group—lack of changes; fasting plasma insulin, HOMA-I andR, HbA1c: unchanged; procyanidins responsible for observed effects (based on extract composition and literature studies)	[148]
*Malus sylvestris* (L.) Mill.	fruits	randomised pre-/post-test; 22 diabetes mellitus type II patients (40–55 years old); 300 g of apple/day (tested) or patients not given fruits (control); duration: 14 days	fasting blood glucose: ↓ by 40.27 ± 23.018 mg/dL in the apple group compared to baseline parameters; in the control group ↓, but not statistically	[150]
*Malus* Mill. sp.	clear or cloudy commercial juice; phlorizin 67/148 µM, chlorogenic acid 378/1304 µM, phloretin xyloglucoside 16/105 µM, D-fructose 6.28/59.3 g/L, D-glucose 22.6/17.9 g/L, sucrose 26.6/27.8 g/L (clear/cloudy juice) (HPLC)	three-way, single-blind, randomised crossover study; nine healthy adults (women and men, 24 ± 3.2 years old); 400 mL of clear or cloudy apple juice (tested) or drink without apple juice (control); duration: acute consumption	plasma glucose: ↓ at 15 and 30 min for clear juice, ↓ at 15 min and ↑ at 45–60 min for cloudy juice, ↓ iAUC (0–30 min), glucose absorption delayed; plasma insulin: ↓ over the first 90 min; GIP: ↓ over the first 90 min, ↓ iAUC (0–30 min); GLP-1: ↑ over the first 90 min (cloudy juice); chlorogenic acid and phlorizin responsible for observed effects (based on extract composition and literature studies)	[152]
*Malus pumila* Mill.; *Pyrus pyrifolia* (Burm.f.) Nakai	fruits; *Malus:* 58% fructose, 30.7% glucose, 11.3% sucrose; *Pyrus:* 57.3% fructose, 36.7% glucose, 6% sucrose;	randomised, eight-period crossover trial; 14 healthy women (18–25 years old); tested: *Malus/Pyrus* + iso-carbohydrate test meals (M + IC or P + IC) or hyper-carbohydrate test meals (M + HC or P + HC); IC, 50 g of available carbohydrate; HC, 65 g of available carbohydrate; 845–1342 kcal; control: water + iso-carbohydrate or hyper-carbohydrate test meals (W + IC or W + HC); duration: acute consumption	*Malus*: ↓ iAUC (0–180 min) by 45.7% and 19.0% for M + IC and M + HC, respectively; ↓ C_max_ by 51.3% and 27.9% for M + IC and M + HC, respectively; maximum amplitude of glycaemic excursion ↓ by about 30–46%; *Pyrus*: ↓ iAUC (0–180 min) by 30.5% and 18.3% for M + IC and M + HC, respectively; ↓ C_max_ by about 40%; maximum amplitude of glycaemic excursion ↓ by about 35–40%	[149]

* Suggestions of what compounds were responsible for the observed effects were taken directly from the cited papers; for a critical discussion, see Section 3.4. ↑, increase; ↓, decrease; C_max_, maximal level; GAE, gallic acid equivalents; GIP, glucose-dependent insulinotropic polypeptide; GLP-1, glucagon-like peptide 1; HbA1c, glycated haemoglobin; HOMA-IR, the homeostasis model assessment-estimated insulin resistance; HPLC, high-performance liquid chromatography; iAUC, incremental area under the curve of tested parameter excursion; OGTT, oral glucose tolerance test; T_max_, time to reach maximal level.

### 3.4. Polyphenols and Other Chemical Contributors to the Anti-Diabetic Activity of Maleae Fruits

**Figure 3:**
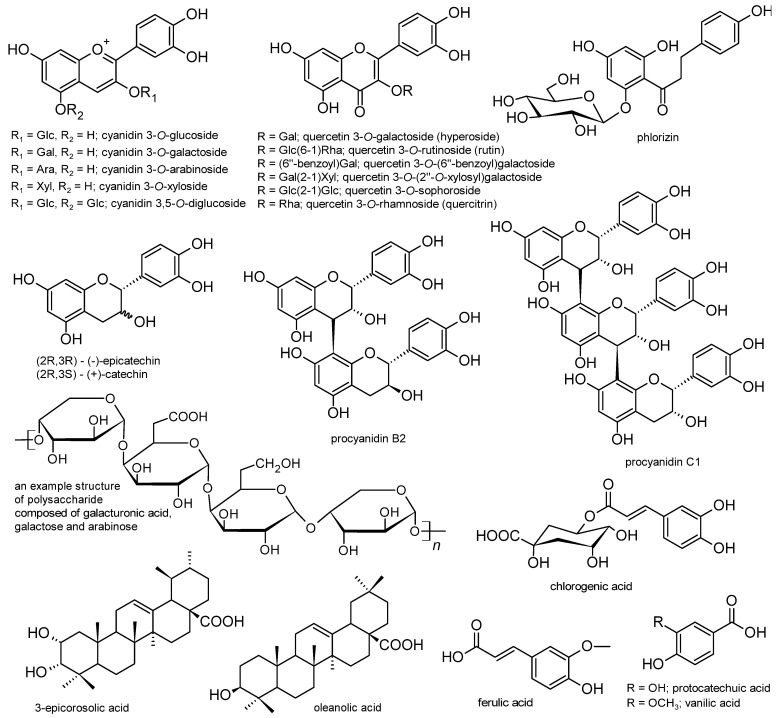
The chemical structures of compounds studied as constituents responsible for the anti-diabetic effects of Malea fruits.

**Figure 4:**
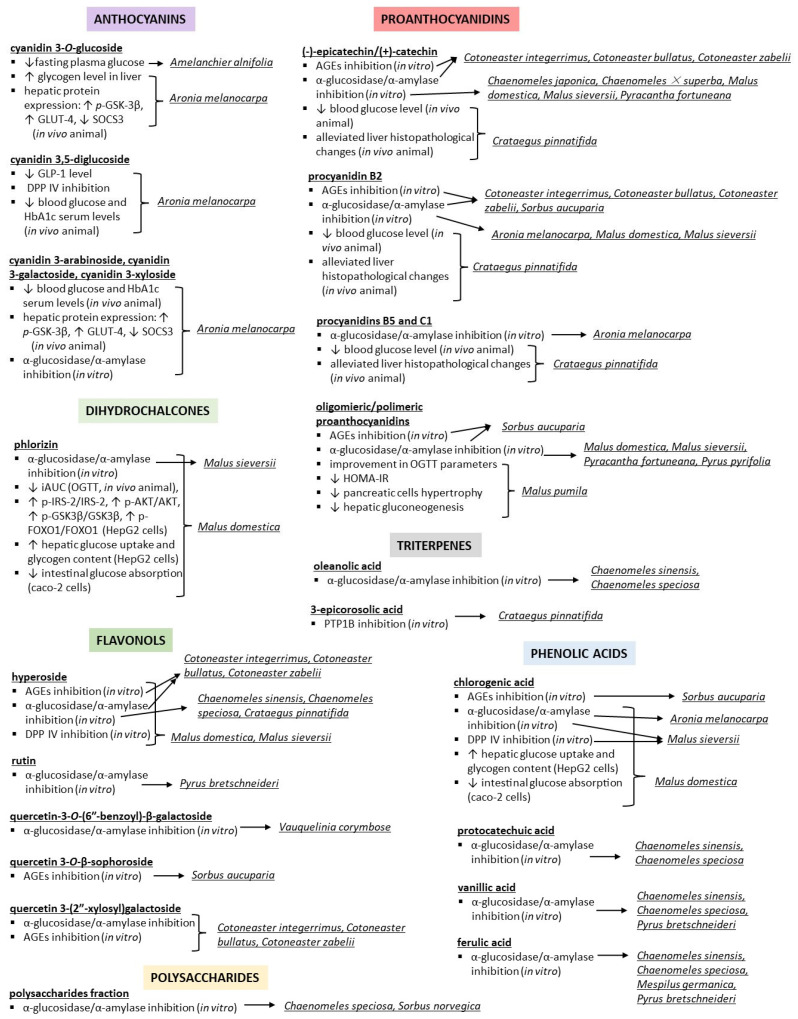
Summarising the contributions of phenolic and non-phenolic compounds to the anti-diabetic effects of Maleae fruits, based on activity studies of pure compounds and their contributions to the fruit samples’ composition and activity [12,14,15,17,20,22,24,27,33,37,42,43,58,59,62,63,64,68,75,79,90,94,100,104,110,113,119]. AGEs, advanced glycation end products; DPP IV, dipeptidyl peptidase-4; GLUT-4, glucose transporter 4; GLP-1, glucagon-like peptide 1; HbA1c, glycated haemoglobin; HOMA-IR, homeostatic model assessment for insulin resistance; OGTT, oral glucose tolerance test; *p*-AKT, phosphorylated protein kinase B; *p*-IRS-2, phosphorylated insulin receptor substrate 2; *p*-FOXO1, phosphorylated forkhead box G1; *p*-GSK-3β, phosphorylated glycogen synthase kinase 3 beta; PTP1B, protein tyrosine phosphatase 1B; SOCS3, suppressor of cytokine signalling 3.

The Maleae fruits contain different bioactive substances, primarily polyphenols (e.g., anthocyanins, flavonoids, proanthocyanidins, phenolic acids), but also terpenoids, proteins, carbohydrates, vitamins, and minerals. In this review, we do not present the detailed chemical composition of all species, since their profiles are highly complex and described in detail in other reviews [153,154,155], but we discuss the suggestions from the literature on the chemical constituents that might be responsible for the observed anti-diabetic effects of fruits. To this end, we focused not on the hypothetical indication of active markers based only on fruit composition (i.e., the presence of particular components), but on activity confirmation by testing pure compounds or their purified fractions (issued in 26 papers). The details, i.a., the chemical structures in question and the brief chemical profiles of the fruits (if covered in the reviewed articles), are shown in Table 1, Table 2 and Table 3. In addition, the impacts of various compounds on the anti-diabetic potential of Maleae fruits are discussed below, and summarised in the form of Figure 3 and Figure 4. All in all, as the leading phytochemical constituents of Maleae fruits, polyphenols were most often tested for biological effects and confirmed as being responsible for the anti-diabetic activity of different species through various mechanisms. In addition, some papers pointed out the contribution of polysaccharides or triterpenes to the biological effects of, e.g., *Chaenomeles* or *Sorbus* species, as well as their synergy with polyphenols.

#### 3.4.1. Anthocyanins’ Contribution to the *Amelanchier* and *Aronia* Fruits’ Activity

Anthocyanins were studied as active constituents of *Amelanchier alnifolia* [104,105,106,107] and *Aronia melanocarpa* [12,58,119]. Cyanidin 3-*O*-glucoside (7.2 mg/kg/day) was revealed to reduce the fasting plasma glucose levels in the mice fed a high-fat, high-sucrose diet, reaching levels similar to those observed for *Amelanchier* berry powder containing an equal amount of cyanidin glucoside [104]. Cyanidin monoglycosides isolated from *Aronia melanocarpa* fruits (986.48 mg of cyanidin galactoside, glucoside, arabinoside, and xyloside per g of fraction; 150–300 mg/kg/day) reduced blood glucose and HbA1c serum levels, increased the glycogen levels in the liver, and modulated hepatic protein expression (↑ *p*-GSK-3β, ↑ GLUT-4, ↓ SOCS3) in the diabetic mice [100]. Moreover, the in vitro studies of the α-glucosidase-inhibitory potential of individual anthocyanins from *Aronia* fruits suggested the higher anti-diabetic potential of cyanidin arabinoside and glucoside (IC_50_ = 0.37–0.87 µg/mL) compared to cyanidin galactoside and xyloside (IC_50_ = 1.54–5.5 µg/mL). Still, all cyanidin monoglycosides were considered to be co-responsible for the anti-glucosidase activity of the chokeberry 50% ethanol extract (IC_50_ = 3.5 µg/mL) [12]. The in vivo (animal model) anti-diabetic potential was also tested for cyanidin 3,5-diglucoside (10 µg/mL solution), but its effectiveness was weaker than that of *Aronia* juice [119]. The contribution of anthocyanins to the anti-diabetic potential of fruits may explain the higher activity observed for extracts from peel than from the flesh of *Amelanchier*, as well as that of acidified alcoholic extracts from *Aronia* fruits compared with non-acidified extracts (higher extraction potential and content of anthocyanins) [13,55].

#### 3.4.2. Dihydrochalcone’s Contribution to the *Malus* Fruits’ Activity

Phlorizin intake (1.96 mg/kg) significantly reduced the iAUC (in vivo animal studies, OGTT) to levels comparable to those achieved by *Malus domestica* commercial extract (12.24 mg/kg) containing the same amount of phlorizin [75]. This observation may be due to the inhibition of intestinal glucose absorption that was reported for phlorizin, e.g., it was calculated that phlorizin contributed to 52% of the glucose transport reduction (Caco-2 cells) noted for *Malus domestica* extract [33]. Phlorizin was also able to inhibit α-glucosidase activity (IC_50_ = 0.01 mg/mL, with significantly stronger effectiveness than extracts from different *Malus sp*. cultivars IC_50_ = 7–256 mg/mL) [42]. Moreover, phlorizin stimulated the glucose uptake by hepatic cells and modified the protein levels in the insulin signalling pathway in vitro, with effects comparable to those achieved with *Malus domestica* 80% ethanolic extract [37].

#### 3.4.3. Flavonols’ Contribution to the *Chaenomeles*, *Cotoneaster*, *Malus*, *Pyrus*, *Sorbus*, and *Vauquelinia* Fruits’ Activity

A contribution to the anti-diabetic potential of Maleae fruits was also suggested for flavonols. Hyperoside was found to inhibit α-glucosidase and/or DPP-IV activity, as well as AGE formation, and the activity was higher than or comparable to that observed for corresponding fruit extracts from *Chaenomeles speciosa, Chaenomeles sinensis, Cotoneaster integerrimus, Cotoneaster zabelii, Cotoneaster bullatus, Crataegus pinnatifida, Malus domestica*, and *Malus sieversii* [15,42,63,68]. Moreover, rutin and quercetin-3-*O*-(6′′-benzoyl)-β-galactoside were suggested to be α-glucosidase inhibitors from *Pyrus bretschneideri* [90] and *Vauquelinia corymbose* [24], respectively, while quercetin 3-(2”-xylosyl)galactoside and quercetin 3-*O*-β-sophoroside contributed to the inhibition of AGE formation observed for *Cotoneaster* sp. [15] and *Sorbus aucuparia* extracts [43], respectively. Finally, it was calculated that quercetin-3-*O*-rhamnoside contributed to the reduction in intestinal glucose transport (in vitro cellular studies on Caco-2 cells) by *Malus domestica* methanol extract, to the extent of 26% [33].

#### 3.4.4. Proanthocyanidins’ Contribution to the *Aronia*, *Crataegus*, *Chaenomeles*, *Cotoneaster*, *Malus*, *Pyracantha*, *Pyrus*, and *Sorbus* Fruits’ Activity

The influence of proanthocyanidins on the anti-diabetic activity of fruits was tested with the use of both purified fractions (composed of monomeric-to-polymeric flavan-3-ols) and individual compounds (i.e., monomers, dimers, and trimers of flavan-3-ols). The four-week supplementation of the procyanidin fraction from *Malus pumila* juice (purified from other active components, like phlorizin and chlorogenic acid, administered without restriction in drinking water, with 0.5 g of procyanidin fraction per 100 mL of water) resulted in a significant improvement in the OGTT parameters and a reduction in HOMA-IR, pancreatic cell hypertrophy, and hepatic gluconeogenesis in insulin-resistant mice [113]. Moreover, the procyanidin fraction from *Crataegus pinnatifida* (epicatechin and procyanidins B2, B5, and C1; 200 mg/kg) reduced blood glucose levels (OGTT) and alleviated histopathological changes to the liver in high-fat-diet rats [110]. Furthermore, different flavan-3-ols isolated from numerous Maleae fruits were proven to inhibit α-glucosidase/α-amylase activity and, thus, suggested to contribute to the fruits’ anti-diabetic activity based on this mechanism; these were (−)-epicatechin/(+)-catechin (*Chaenomeles* sp., *Cotoneaster* sp., *Malus* sp.), procyanidin B2 (*Aronia melanocarpa, Cotoneaster* sp., *Malus* sp., *Sorbus aucuparia*), procyanidin C1 (*Aronia melanocarpa*), oligomeric and polymeric proanthocyanidin fractions (*Pyrus pyrifolia, Sorbus aucuparia*), the sum of (+)-catechin, (−)-epicatechin, A-type and B-type procyanidins, and procyanidin glycosides (*Pyracantha fortuneana*) [12,15,20,27,42,43,63,64,79,96]. In addition, (−)-epicatechin and procyanidin B2 were demonstrated to inhibit AGE formation, and their effectiveness was a dozen to several dozen times higher than that observed for corresponding fruit extracts from *Cotoneaster* sp. and *Sorbus aucuparia* [15,43].

#### 3.4.5. Phenolic Acids’ Contribution to the *Aronia*, *Chaenomeles*, *Malus*, *Mespilus*, *Pyrus*, and *Sorbus* Fruits’ Activity

Regarding phenolic acids, their contribution to the anti-diabetic activity of Maleae fruits was best documented for chlorogenic acid. It was reported that chlorogenic acid contributed to the inhibition of intestinal glucose transport (observed for the *Malus domestica extract*, 12% share [33]), the inhibition of α-glucosidase activity (*Aronia melanocarpa* wines, *Chaenomeles sp*. extracts, and *Malus* sp. extracts [14,42,63]), the inhibition of hepatic glucose uptake and DPP-IV activity (*Malus* sp. extracts [37,42]), and the inhibition of AGE formation (*Sorbus aucuparia* extracts [43]). Moreover, the reduction in α-glucosidase activity was also observed for protocatechuic, ferulic, and vanillic acids from *Chaenomeles* sp., *Mespilus germanica*, and *Pyrus bretschneideri* extracts [63,87,90].

#### 3.4.6. Contribution of Non-Phenolic Compounds to the *Crataegus*, *Chaenomeles*, and *Sorbus* Fruits’ Activity, and Their Synergy with Polyphenols

Apart from polyphenols, some triterpenes were also suggested as anti-diabetic agents. For instance, 3-epicorosolic acid isolated from *Crataegus pinnatifida* inhibited protein tyrosine phosphatase 1B (PTP1B) and α-glucosidase to comparable or higher extents than the respective crude methanol extract and its organic fractions [17]. In addition, α-glucosidase-inhibitory activity was observed for oleanolic acid from *Chaenomeles* sp. [59,63].

Last, but not least, the anti-diabetic potential has been confirmed for polysaccharides. The α-glucosidase inhibition observed for the polysaccharide fraction from *Chaenomeles speciosa* fruits was potent (100% inhibition at 0.5 mg/mL) and significantly higher compared to all other compounds isolated from the fruits, including various polyphenols and triterpenes. However, there was no clear relationship between activity parameters and polyphenol/polysaccharide/triterpene concentrations in different *Chaenomeles* extracts/fractions. Consequently, statistical analysis performed using composition data of the tested extracts/fractions and activity of pure compounds suggested that α-glucosidase inhibition of the fruits seems additive or synergic and depends on various chemical constituents and proportions between them [59]. The synergy of polysaccharides with polyphenols in the context of anti-diabetic potential was also confirmed for *Sorbus norvegica*. In this case, the inhibition of α-amylase activity was significantly higher for the whole-fruit extract (IC_50_ = 2.5 µg/mL) than for its two fractions, i.e., polysaccharides (IC_50_ = 48 µg/mL) and polyphenols (IC_50_ = 20 µg/mL) [22]. The structures of both tested polymers were not analysed. Still, another study on the carbohydrates from *Chaenomeles* and *Sorbus* species suggested that galacturonic acid, arabinose, and galactose may be the primary components of these active polysaccharides [156,157].

#### 3.4.7. Impact of Monosaccharides on Fruits’ Anti-Diabetic Potential

Considering the possibility of using fruits as potential herbal drug candidates for the prevention or treatment of diabetes, it is worth paying attention to the presence of simple sugars and the related glycaemic index (GI). The GI expresses the capacity of the organism to deal with carbohydrates in foods as a percentage of the response to an equal weight of glucose. Since all fruits from the Maleae tribe are classified as having a low GI (<55), their consumption by diabetic patients is considered to be safe [158,159,160]. This is especially important for ingesting the whole, fresh fruits or juices, while supplementation of fruit extracts with concentrated contents of only selected compounds is all the more secure.

### 3.5. The Anti-Diabetic Potential of the Most Promising Maleae Fruits—Concluding Thoughts

Considering all studies on the anti-diabetic effects of Maleae fruits, their number, and the results presented above, the most promising species for more expansive pro-health applications today seem to be *Aronia melanocarpa* and *Malus domestica*, which are among the few that have been tested in human trials. Therefore, in this chapter, we sum up the currently available data on these two species and discuss the potential outcomes for future diabetes management using fruit products. At the same time, we do not forget about other species, research on which is less advanced but still promising and worthy of further attention, e.g., *Amelanchier* sp., *Chaenomeles* sp., *Crataegus* sp., *Pyrus* sp., *Sorbus* sp., and other *Aronia* sp., and *Malus* sp.

#### 3.5.1. The Anti-Diabetic Potential of *Aronia melanocarpa* Fruits

The anti-diabetic potential of *Aronia melanocarpa* seems to be mainly due to the increase in insulin sensitivity and the boost in the peripheral absorption of glucose (likewise, e.g., metformin), as well as the delay in the absorption of carbohydrates from the intestine (likewise, e.g., acarbose).

According to Chen and Meng [100], the five-week supplementation of acidified 80% ethanol *Aronia melanocarpa* fruit extract, at 300 mg/kg/day, resulted in about 1.6-fold lower glucose levels, about 2-fold lower insulin levels, and about 1.8-fold lower HbA1c levels in comparison to diabetic mice, while the effect of metformin (200 mg/kg/day) was about 2-fold lower glucose levels, about 2.2-fold lower insulin levels, and about 2.3-fold lower HbA1c levels. The effectiveness of *Aronia* extract relative to the synthetic anti-diabetic drug was also observed in the enhancement of GLUT-4 and p-GSK-3β expression, resulting in higher glucose uptake by hepatic cells and stimulating glycogen synthesis [100]. The increased tissue-specific glucose uptake and glycogen levels, as well as the modulation of different proteins’ expression in the insulin signalling pathway, which led to a decrease in glucose levels, was confirmed by many in vitro and in vivo studies (Table 1, Table 2 and Table 3). What is essential in this metformin-like mechanism of action is that the sensitisation of tissues to the action of insulin occurs without increasing the insulin level, which reduces the risk of hypoglycaemia as the main disadvantage of sulfonylurea drugs (e.g., glipizide).

The delay in the absorption of carbohydrates from the intestine observed for *Aronia melanocarpa* fruit products relies on glucosidase inhibition, which is therefore another verified mechanism of action of the fruit. According to different studies [114,115], *Aronia* juices and extracts significantly inhibited α-glucosidase, maltase, and sucrase activity in the intestine (in vivo animal models). The effectiveness of *Aronia* extracts towards α-glucosidase compared to acarbose (in vitro studies) was measured as 37–186 times higher, depending on the fruit cultivar, the origin of the sample, and the type of extract (and, thus, chemical composition). It is also advantageous that synthetic glucosidase inhibitors are associated with some slight gastrointestinal side effects, of which fruits seem to be devoid [143,144].

Finally, as mentioned above, the activity of fruit products is closely related to their composition. From the whole chemical pool of *Aronia melanocarpa* fruits (over one hundred phenolic compounds identified), only a few have been tested as contributors to the anti-diabetic potential of fruit. These were mainly anthocyanins (i.e., cyanidin 3-arabinoside, cyanidin 3-glucoside, cyanidin 3-galactoside, cyanidin 3-xyloside, cyanidin 3,5-diglucoside), which accounted for about 10–25% (or 98% in case of the purified fraction) of extracts/juice dry mass (Table 1, Table 2 and Table 3). Like *Aronia* fruits, these compounds were able to lower blood glucose, HbA1c, and GLP-1 levels, inhibit DPP IV and α-glucosidase activity, increase glycogen levels in the liver, and modulate hepatic protein expression (in vitro and in vivo models) [12,58,100,119]. The contribution to the α-glucosidase-inhibitory activity was also noticed for procyanidin dimers, trimers, and some phenolic acids (i.e., chlorogenic and caffeic acids) [12,14,58]. Moreover, considering the contents of individual compounds and their activity, as well as the activity of relevant plant samples, the synergistic and additive effects of different constituents were suggested [119,131]. Synergy with synthetic anti-diabetic drugs is also possible, but this matter has not been studied so far.

#### 3.5.2. The Anti-Diabetic Potential of *Malus domestica* Fruits

In the case of *Malus domestica* fruits, their effectiveness is suggested to be mainly due to the inhibition of both sodium-dependent and sodium-independent glucose transporters in the intestine (SGLT-1 and GLUT-2), which results in a delay in the absorption of carbohydrates [32,34]. SGLT-1 inhibitors are currently an eagerly studied group of anti-diabetic drugs (synthetic and plant-derived) whose potential is due not only to their glucose-lowering ability but also to their cardioprotective effects. One of the leading natural SGLT-1 inhibitors is phlorizin—a dihydrochalcone isolated from apples [147]. Indeed, the phlorizin-containing apple products were also confirmed to reduce glucose-derived protein damage by inhibiting the activity of ALR and SDH enzymes and the formation of AGEs (polyol pathway); thus, they may prevent the development of diabetic complications [41,74,80].

The second type of *Malus domestica* fruit activity related to the content of phlorizin (5–16%) was suggested to be the increased insulin sensitivity resulting from the enhanced hepatic glucose uptake and expression of proteins involved in glycogen synthesis and glycolysis (e.g., ↑ *p*-GSK3β/GSK3β, ↑ *p*-FOXO1/FOXO1) [147]. Therefore, *Malus domestica* is another species with a metformin-like mechanism of action, i.e., the sensitisation of tissues to the action of insulin without increasing the insulin level. Indeed, the in vivo studies confirmed the *Malus* extracts’/juices’ ability to lower both glucose and insulin in diabetic animals to levels comparable to those observed in healthy subjects, as well as to normalise the postprandial and OGTT parameters in healthy people [32,34,73,75,109,139,140,143].

As for anti-diabetic bioactive compounds, aside from phlorizin, there has also been research on the contributions of chlorogenic acid, procyanidins, and quercetin derivatives to hepatic and intestinal glucose transport, as well as AGEs and α-glucosidase inhibition [33,37,42,79]. Interestingly, while the inhibitory potential of apples towards α-glucosidase seems to be less evident (i.e., only extracts with increased contents of polyphenols—about 40–80%—were able to inhibit glucosidase more strongly than acarbose), the synergistic anti-glucosidase activity with acarbose was confirmed for apple juice. Thus, this is another beneficial aspect of using fruit products in combinatory therapy for diabetes.

## 4. Conclusions

The data reported in this review show that many Maleae fruits indeed have anti-diabetic potential and may be recommended for the prevention and treatment of diabetes. However, from over 1000 species belonging to the Maleae tribe, only 46 have been investigated so far in the context of diabetes. The majority of the conducted studies covered only in vitro tests (67 papers); then, there are in vivo studies on animal models (51 reports, including some mixed in vitro/in vivo tests), as well as in vivo human studies (14 trials on *Aronia melanocarpa, Malus* sp., and *Pyrus pyrifolia*). The species most thoroughly studied in terms of anti-diabetic effects and mechanisms were *Amelanchier alnifolia*, *Aronia melanocarpa, Chaenomeles japonica, Crataegus pinnatifida, Malus domestica, Malus pumila,* and *Pyrus pyrifolia.* The reviewed papers indicated the ability of Maleae fruits, e.g., to modulate the expression and activity of the proteins in the insulin-mediated PI3K/Akt pathway, induce incretin-based effects (e.g., GLP-1 and GIP agonism, DPP IV inhibition), regulate the intestinal glucose absorption and tissue-specific glucose uptake by affecting the glucose transporters (GLUTs and SGLT1), and inhibit enzymes involved in carbohydrate digestion (α-glucosidase) or in the polyol pathway of glucose metabolism (ALR, SDH). As for phytochemicals responsible for the anti-diabetic effectiveness of Maleae fruits, some reviewed papers suggested contributions of various compounds to the observed effects—primarily polyphenols (e.g., flavonols, dihydrochalcones, proanthocyanidins, anthocyanins, phenolic acids), but also triterpenes and polysaccharides. Thanks to the additive and synergistic actions of individual phytochemicals, the biological effects of fruits/juices/extracts are significantly higher than those of pure compounds. Therefore, various fruits from the Maleae tribe seem to be advantageous anti-diabetic agents. Still, their potential for functional application depends on various factors, in addition to the obvious plant species/variety, all of affect the composition of fruit products and, thus, may explain some discrepancies in the results of different studies. These include variations in climatic growth conditions, maturity stage, type of sample (i.e., the part of the fruit, like peel or flesh), processing conditions, or ingested form, e.g., if consumed as fresh fruits, juices, or specific extracts with high concentrations of selected active compounds. The latter form seems especially promising, as it reduces excessive intake of ballast constituents, primarily diabetes-promoting free monosaccharides. Finally, the prospects of different fruits and their extracts for more expansive pro-health applications require further research, especially more profound in vivo trials with the establishment of effective doses, and formulation and toxicity studies on the fruit extracts as potential herbal drug candidates.

## Data Availability

Not applicable.
